# Identification of DNA methylation characteristics associated with metastasis and prognosis in colorectal cancer

**DOI:** 10.1186/s12920-024-01898-4

**Published:** 2024-05-10

**Authors:** Fang Qian, Qiang Li, Huidan Chang, Kai Wei, Xiaoyi Chen, Tao Huang, Yixue Li

**Affiliations:** 1 Bio-Med Big Data Center, CAS Key Laboratory of Computational Biology, Shanghai Institute of Nutrition and Health, University of Chinese Academy of Sciences, Chinese Academy of Sciences, Shanghai, 200031 China; 2 Guoke Ningbo Life Science and Health Industry Research Institute, Ningbo, 315000 China; 3 Key Laboratory of Systems Health Science of Zhejiang Province, School of Life Science, Hangzhou Institute for Advanced Study, University of Chinese Academy of Sciences, Hangzhou, 310024 China; 4 Guangzhou Laboratory, Guangzhou, 510005 China; 5 School of Life Sciences and Biotechnology, Shanghai Jiao Tong University, Shanghai, 200240 China; 6 Collaborative Innovation Center for Genetics and Development, Fudan University, Shanghai, 200433 China

**Keywords:** Colorectal cancer (CRC), DNA methylation, Tumor immune microenvironment (TME), Tumor metastasis, Prognosis

## Abstract

Colorectal cancer (CRC) is prone to metastasis and recurrence after surgery, which is one of the main causes for its poor treatment and prognosis. Therefore, it is essential to identify biomarkers associated with metastasis and recurrence in CRC. DNA methylation has a regulatory role in cancer metastasis, tumor immune microenvironment (TME), and prognosis and may be one of the most valuable biomarkers for predicting CRC metastasis and prognosis. We constructed a diagnostic model and nomogram that can effectively predict CRC metastasis based on the differential methylation CpG sites (DMCs) between metastatic and non-metastatic CRC patients. Then, we identified 17 DMCs associated with progression free survival (PFS) of CRC and constructed a prognostic model. The prognosis model based on 17 DMCs can predict the PFS of CRC with medium to high accuracy. The results of immunohistochemical analysis indicated that the protein expression levels of the genes involved in prognostic DMCs were different between normal and colorectal cancer tissues. According to the results of immune-related analysis, we found that the low-risk patients had better immunotherapy response. In addition, high risk scores were negatively correlated with high tumor mutation burden (TMB) levels, and patients with low TMB levels in the high-risk group had the worst PFS. Our work shows the clinical value of DNA methylation in predicting CRC metastasis and PFS, as well as their correlation with TME, immunotherapy, and TMB, which helps understand the changes of DNA methylation in CRC metastasis and improving the treatment and prognosis of CRC.

**Supplementary Information:**

The online version contains supplementary material available at 10.1186/s12920-024-01898-4.

## Background

Colorectal cancer (CRC) is a common and highly invasive tumor in the digestive tract, and its incidence rate and mortality are increasing yearly [1]. CRC is prone to metastasis or recurrence after conventional treatment (such as surgical resection), and the 5-year survival of CRC patients with metastasis is worse than that of primary cancer patients [2]. At present, systemic chemotherapy is often used to inhibit the growth and spread of cancer cells. However, it cannot eliminate potential disseminated cancer cells and only benefits patients for several months [3]. Cancer metastasis seriously affects the treatment and survival of CRC patients [4]. Therefore, it’s urgent to understand the process of metastasis and screen new biomarkers that can predict the metastasis and prognosis of CRC patients.

Accumulative evidence shows that abnormal DNA methylation regulates cancer occurrence and progression [5]. The change of DNA methylation pattern occurs in the early stage of carcinogenesis and leads to the silencing of multiple tumor suppressor genes in CRC [6]. It is reported that DNA methylation may directly affect gene transcription to promote cancer transformation and tumor metastasis [7, 8]. In addition, several studies have been conducted to find abnormal methylation biomarkers based on DNA in plasma or feces to develop noninvasive diagnostic tools related to CRC [9]. Jin et al. developed a quantitative analysis method for DNA methylation markers to monitor CRC [10] effectively. Therefore, DNA methylation-related features may become a promising candidate for CRC biomarker development.

Many epigenetic studies have confirmed that DNA methylation plays a key regulatory role in inflammation, TME, and immunotherapy [11]. Xu et al. have shown that DNA methylation profiles can predict immunotherapy responses at the pan-cancer level [12]. Based on the 24 DNA methylation regulators in CRC, Yuan et al. distinguished 3 DNA methylation patterns with different TME and prognostic features [13]. Therefore, it is of great importance to investigate the correlation between DNA methylation with TME in guiding immunotherapy and improving the prognosis of CRC.

In this study, we elucidated the changes in DNA methylation during CRC metastasis and its correlation with prognosis and TME. Firstly, we identified differentially methylated CpG sites (DMCs) between CRC metastatic and non-metastatic groups. Subsequently, DMCs that can predict CRC transfer were identified. Then, we constructed a DNA methylation-related prognosis and nomogram model that can predict PFS in CRC patients to evaluate the clinical value of metastasis-related DMCs. In addition, the correlation of DNA methylation with TME and immunotherapy in CRC was determined by immune infiltration correlation analysis, MSI analysis, and tumor mutation burden (TMB) analysis.

## Materials and methods

### Data acquisition and preprocessing

The 450 K DNA methylation array (*n* = 289), 27 K DNA methylation array (*n* = 153) of colorectal cancer (CRC) patients, and the corresponding clinical information and progression free survival data were obtained from the UCSC Xena database. 27 K DNA methylation array was used for the validation dataset of the prognosis model. Patients in the TCGA-COAD queue have received various treatment methods, including surgery, radiation therapy, drug therapy, and immunotherapy. CRC samples with progression free survival times greater than 0 and their corresponding data were used for subsequent analysis. 450 K DNA methylation array (GSE164811) of CRC patients was obtained from GEO database as the validation dataset of the diagnostic model. In addition, we downloaded tumor mutation burden (TMB) data related to CRC patients from TCGA database.

Then, quality control of CpG sites in the DNA methylation array was performed. First, remove the CpG locus located on the sex chromosome. Remove CpG sites null in more than 70% of the samples, and retain CpG sites where the transcription start site is 2 kb upstream to 0.5 kb downstream. The overlapping CpG sites in the 450 K DNA methylation array, 27 K DNA methylation array in TCGA, and 450 K DNA methylation array in GEO were reserved. Finally, 21,122 CpG sites were retained. Subsequently, KNN was used to impute missing values in DNA methylation array data. The “SVA” package was utilized to remove the batch effect between the three DNA methylation arrays.

### Differential analysis of methylation CpG sites

In this study, CRC patients were separate the tumor metastasis group and tumor non-metastasis group according to M (distant metastasis) stage and N (lymph node metastasis) stage. CRC patients with both M stage and N stage 0 (M = M0 & N = N0) were included in the tumor non-metastasis group, and the remaining CRC patients were included in the tumor metastasis group. In order to obtain differential methylation CpG sites (DMCs) in patients with and without metastasis, we used the “ChAMP” package to standardize and analyze the difference of 450 K DNA methylation array in TCGA. The methylation CpG sites with *P* values less than 0.01 were considered to be significantly different. These DMCs intersect with 21,122 methylation sites obtained by QC as the final DMCs.

### Functional enrichment analysis

We annotated the genes where DMCs are located. We conducted functional enrichment analysis to clarify the biological processes involved in developing CRC by DMCs. The “clusterProfiler” package was used to perform KEGG analysis. The condition of significant pathway is that its *p* value is less than 0.05. Next, we conducted Metascape analysis on the online Metascape platform (https://metascape.org/) for the genes where DMCs are located.

### Identification of DMCs associated with tumor metastasis in CRC

Firstly, univariate Cox regression analysis and KM test were conducted to evaluate the correlation between DMCs expression and PFS of CRC. DMCs with *p* values less than 0.05 were reserved for subsequent analysis. R packages “igraph” and “reshape2” were utilized to map the network of PFS-related CpG sites. The SVM-RFE (R package “e1071”) method was used to identify DMCs associated with metastasis in CRC. An SVM classifier based on the β values of DMCs was constructed to predict CRC metastasis. The random seed was set to "124579". ROC curve was used to evaluate the accuracy of the diagnostic model constructed based on DMCs in predicting CRC metastasis. Based on the CRC metastasis-related DMCs obtained from SVM screening, we constructed a nomogram using the “rms” package. The calibration curve was utilized to evaluate the accuracy of the nomogram in predicting metastasis in CRC patients. Decision Curve Analysis (DCA) and Clinical Impact Curve were used to assess the performance of the nomogram.

###  Construction of prognosis model and nomogram related to the PFS of CRC

To further identify CpG sites associated with PFS in CRC patients, LASSO-Cox regression analysis (“glmnet” package) was conducted on DMCs in the diagnostic model. Through 1000 iterations, the optimal penalty parameter λ of the model was determined. The risk score of each CRC patient was obtained based on the β values (${CpG}_{i}$) of the candidate CpG sites and their corresponding regression coefficients (${coef}_{i}$). $\text{r}\text{i}\text{s}\text{k}\text{S}\text{c}\text{o}\text{r}\text{e}=\sum\nolimits_{i=1}^{n}\left({CpG}_{i}*{coef}_{i}\right)$, n is the number of methylated CpG sites associated with PFS in CRC patients. Next, Kaplan-Meier analysis was performed to determine the difference in PFS between the two risk groups. The ROC curve was used to evaluate the accuracy of the constructed prognostic model in predicting 1- and 3-year PFS of CRC patients. Independent prognostic analysis was performed on the clinical characteristics of CRC as well as the risk scores of prognostic models to identify factors that could independently predict PFS in CRC patients.

The “rms” package was conducted to construct a nomogram for predicting PFS of CRC patients. The calibration curve and ROC curve were drawn to evaluate the performance of the nomogram model in predicting the PFS of CRC patients.

### Immune microenvironment and microsatellite instability analysis

We utilized ssGSEA to obtain the scores of immune cells and immune-related pathways in CRC patients. In addition, we performed immune correlation analysis on the DNA methylation data (450 K) of TCGA-COAD using the HiTIMED algorithm. In order to determine the sensitivity of CRC patients in high- and low-risk groups to immunotherapy, we calculated the tumor immune dysfunction and exclusion (TIDE) score of the TCGA-COAD dataset from the TIDE online analysis platform (http://tide.dfci.harvard.edu/). The higher the TIDE score, the worse the immune response of patients. In addition, we conducted microsatellite instability (MSI) analysis to explore the correlation between risk score and MSI.

###  Tumor mutation burden analysis

The “maftools” package was utilized to draw waterfall diagrams to show the TMB levels of two risk subgroups. In order to further determine the correlation between TMB and PFS of CRC patients, KM analysis was used to compare the difference of PFS of CRC patients between high- and low-TMB groups.

### Immunohistochemical analysis

To further elucidate the role of DMCs in the progression of CRC, we explored the expression at the protein level of genes in which prognostic related DMCs are located. We downloaded the immunohistochemical maps of normal and CRC tissues from the Human Protein Atlas (HPA) database.

### Statistical analysis

**Fig. 1:**
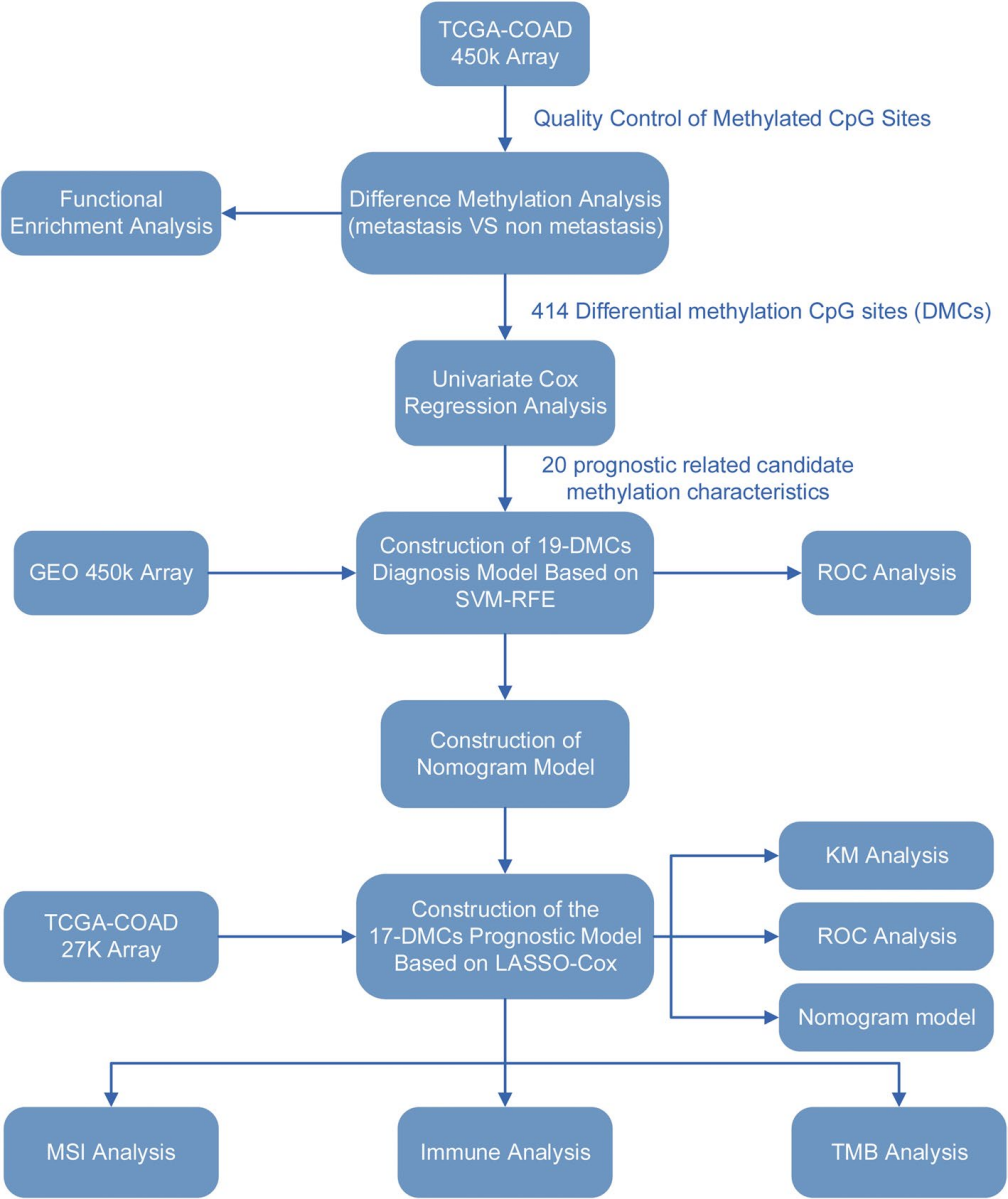
Workflow of the research

The data analysis and results visualization of this study was conducted on R (4.2.2). T-test or Wilcoxon-test was used to compare the differences between groups. Spearman was used for correlation analysis. In this study, a *p*-value less than 0.05 is statistically significant, and “*” represents *p* < 0.05, “**” represents *p* < 0.01, and “***” represents *p* < 0.001. The workflow is shown in Fig. 1.

## Results

### Identification of differential methylated CpG sites

**Fig. 2:**
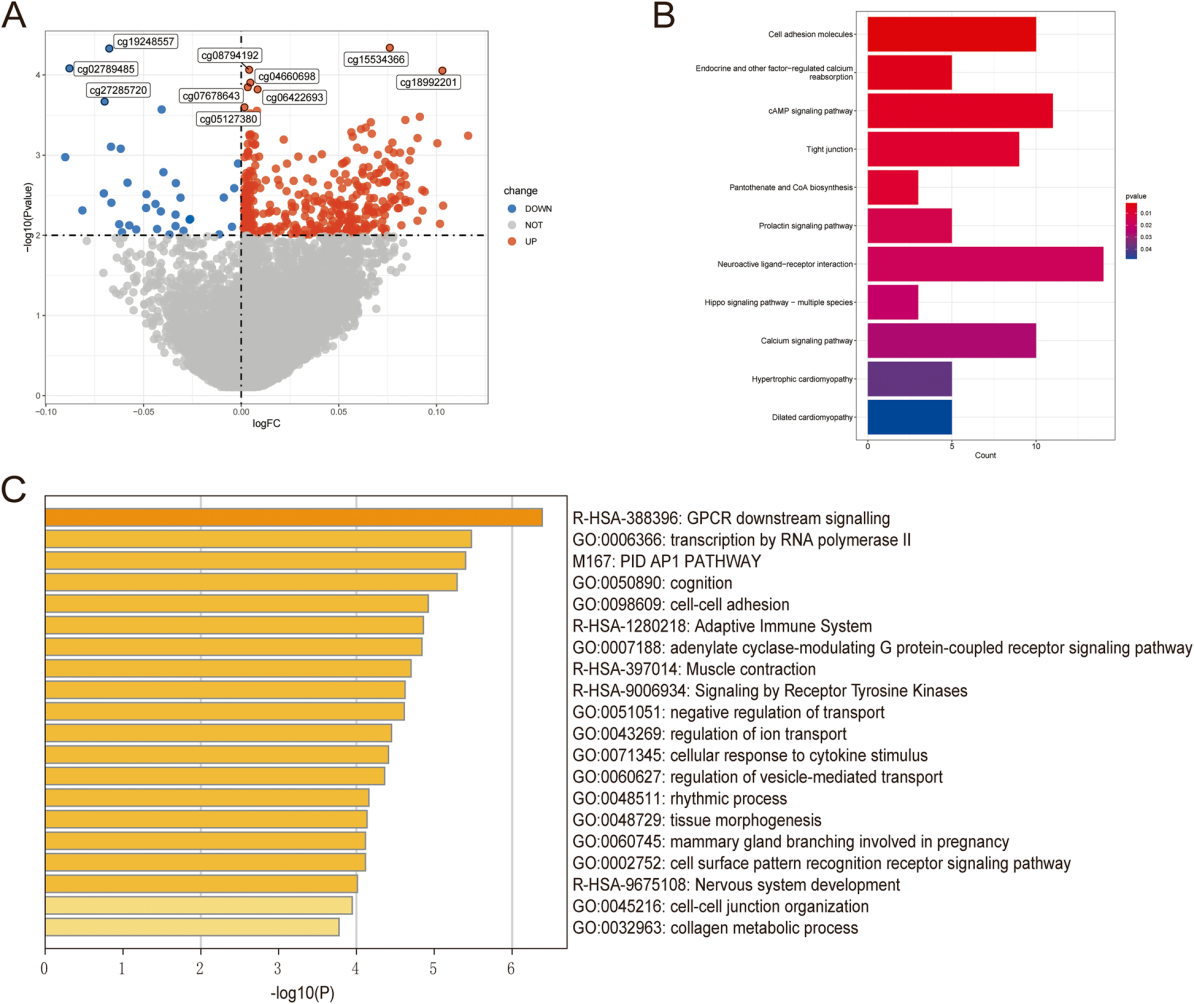
Identification of differential methylation CpG Sites (DMCs). **A** Volcano plot of DMCs between metastatic and non-metastatic CRC patients. The red represents the up-regulated methylation CpG Sites, blue represents the down-regulated methylation CpG Sites. **B** KEGG analysis of DMCs located genes. **C** Metascape analysis of DMCs located genes

According to TCGA-COAD 450 K methylation data, according to the *p*-value less than 0.01, there are 42,310 methylation CpG sites with differences between the metastatic and non-metastatic groups (Supplementary material 1). Then, 42,310 CpG sites obtained by the differential analysis were intersected with 21,122 CpG sites after quality control, and 414 CpG sites were used as DMCs for subsequent analysis. We found that the β values of DMCs were upregulated more in metastatic CRC patients. Among them, 380 DMCs had higher β values in the metastasis group than in the non-metastasis group, and 34 DMCs had lower β values in the metastasis group (Fig. 2A). In Fig. 2B, the genes where DMCs are located were mainly participate in ligand-receptor interaction, Tight junction, Neuroactive cAMP signaling pathway, Cell adhesion molecules, and other biological pathways (Supplementary material 2). Metasape analysis showed that the genes where DMCs are located are mainly involved in GPCR downstream signalling, transcription by RNA polymerase II, PID AP1 PATHWAY, and cognition (Fig. 2C, Supplementary material 3).

### Methylation characteristics associated with CRC metastasis

**Fig. 3:**
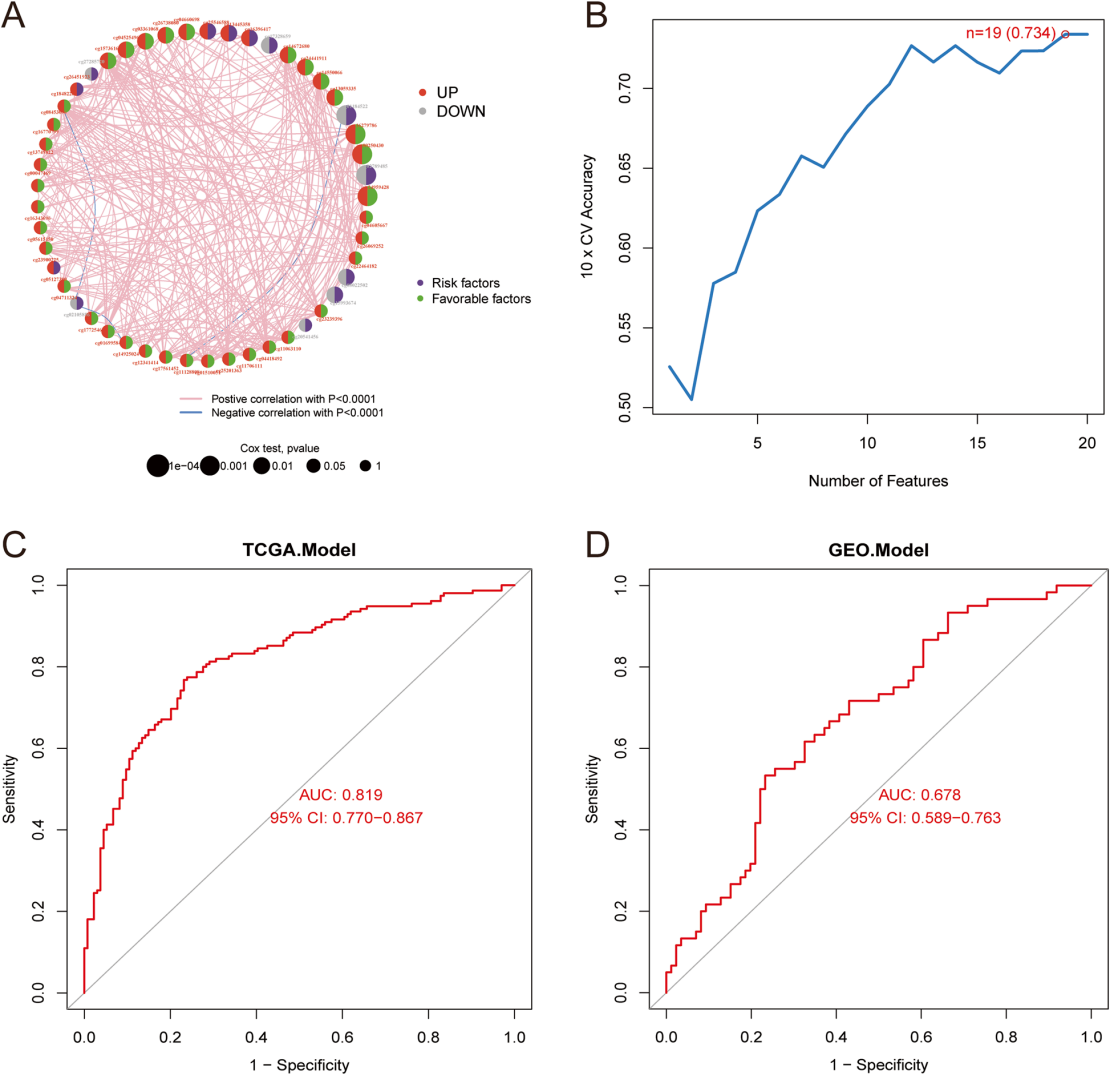
Construction of diagnostic model for predicting CRC metastasis. **A** Circos graph for univariate Cox regression analysis, which represents the correlation of DMCs β value and prognosis of CRC (red: up-regulated methylation CpG Site, grey: down-regulated methylation CpG Site, purple: risk factor; green: favorable factor) in the TCGA-COAD (*p*-values for Cox test: 1e- 04 to 1). **B** Line plot of 10-fold cross-validation of the SVM-RFE algorithm for feature selection. ROC curves of diagnostic model in the TCGA cohort (**C**) and GEO cohort (**D**)

In order to screen CpG sites that could diagnose metastasis in CRC patients, we performed a comprehensive analysis. First, we utilised univariate Cox regression analysis and KM test to discern candidate DMCs associated with PFS in CRC patients. Among them, 186 DMCs with a *p*-value less than 0.05 in the KM test (Supplementary material 4). In Fig. 3A, we plotted the network of the top 50 CpG sites interactions, expression, and their association with PFS in CRC patients. DMCs with *p* values less than 0.05 in univariate Cox regression analysis and KM test were used to construct a diagnostic model for predicting metastasis of CRC patients, and a total of 20 DMCs were obtained. Subsequently, we used SVM-RFE to screen methylation signatures predicting metastasis in CRC patients based on the β values of 20 DMCs. According to the results of 10-fold cross-validation (Fig. 3B), the diagnostic model had the highest accuracy (0.734) when the number of methylation features was 19 (cg04660698, cg02789485, cg03361068, cg26738080, cg25546588, cg14550066, cg08022502, cg17328659, cg01184522, cg15993674, cg24441911, cg04525496, cg14672680, cg13445358, cg15736165, cg16279786, cg16396417, cg00250430, and cg13059335). The results of ROC analysis declared that 19 DMCs diagnostic model had high accuracy in predicting the metastasis of CRC patients in the TCGA-COAD cohort (Fig. 3C, AUC = 0.819) and the GEO cohort (Fig. 3D, AUC = 0.678).

**Fig. 4:**
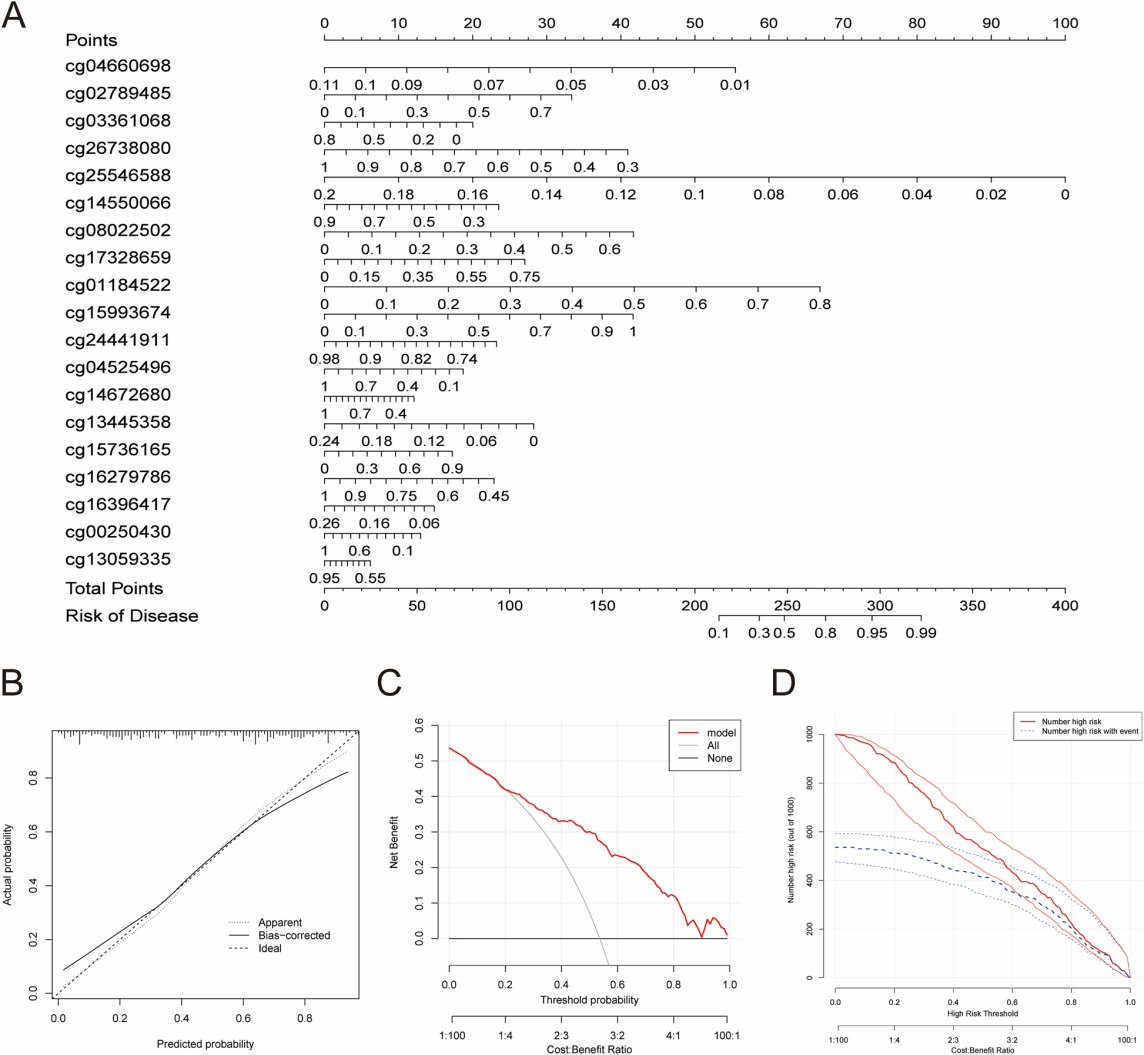
Construction of a nomogram model for predicting CRC metastasis based on the TCGA cohort. **A** Nomogram to predict the metastasis of CRC. **B** Calibration curve to assess the predictive power of the nomogram model. DCA curve (**C**) and clinical impact curve (**D**) to evaluate the clinical value of the nomogram model

Then, we constructed a nomogram model based on the DMCs in the diagnostic model to predict the risk of metastasis in CRC patients (Fig. 4A). In Fig. 4B, the calibration curve showed that the error between the actual risk of CRC patients and the metastasis risk predicted by the nomogram is minor, suggesting that the nomogram based on 19-DMCs can predict the metastasis of CRC with high accuracy. DCA analysis showed that compared with the all or no patient intervention scheme, CRC patients would benefit more from using this nomogram to predict the probability of metastasis at a high risk threshold (0–1), which indicated that the clinical application of 19-DMCs nomograms has a higher impact (Fig. 4C). The clinical impact curve showed that the “Number high risk” curve and the “Number high risk with event” curve were close to 1 from 0.3, which revealed that the nomogram had a good predictive ability (Fig. 4D). The results suggested that these 19 DMCs may play an essential role in the metastasis of CRC.

### Identification of PFS related methylation signatures in CRC

**Fig. 5:**
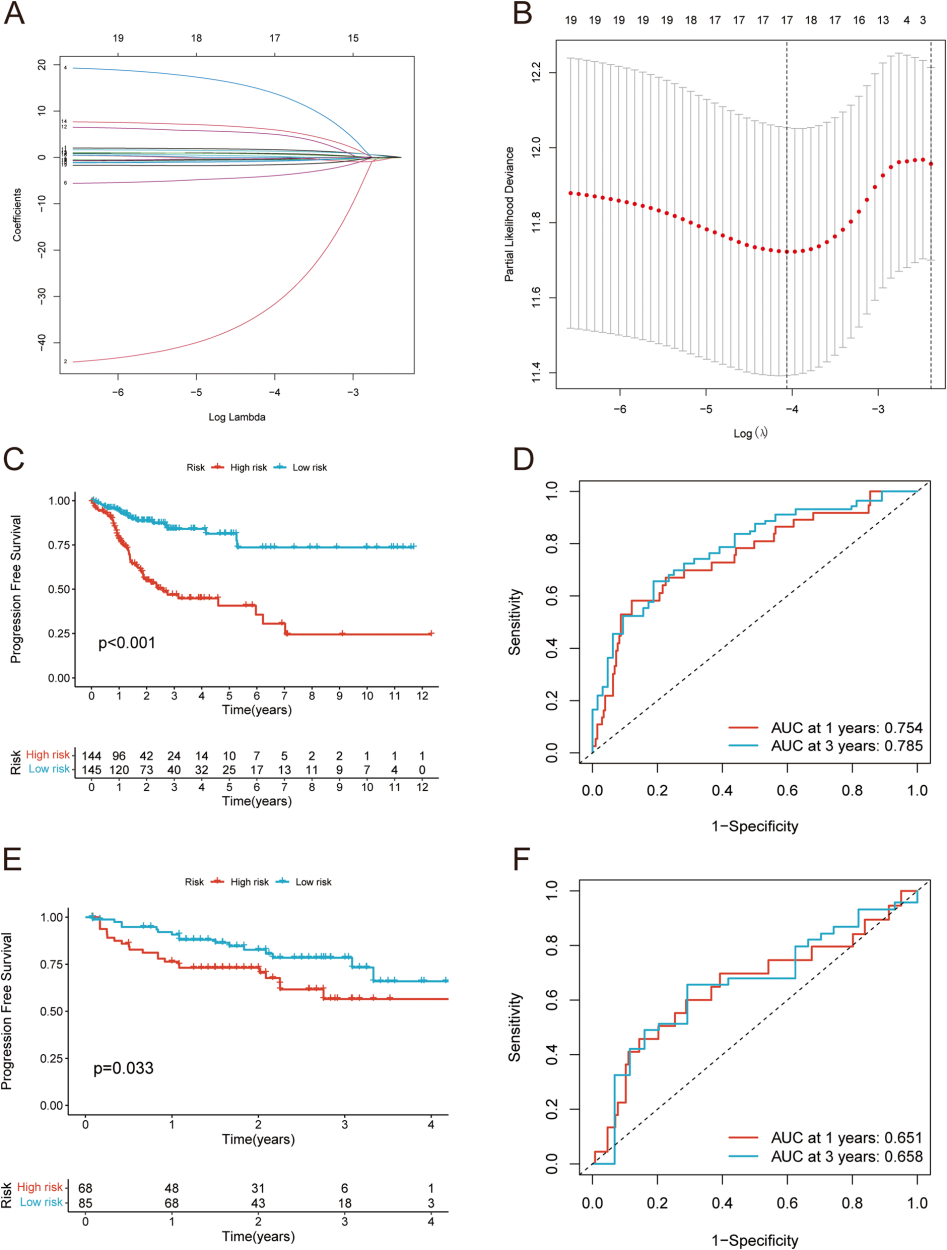
Establishment of a 17-DMCs signature. **A** and **B** LASSO Cox regression (with minimized lambda) of the DMCs. Survival curve showing different PFS between high- and low-risk groups in training dataset (**C**) and testing dataset (**E**). 1-year and 3-year ROC curves for training dataset (**D**) and testing datasets (**F**)

**Fig. 6:**
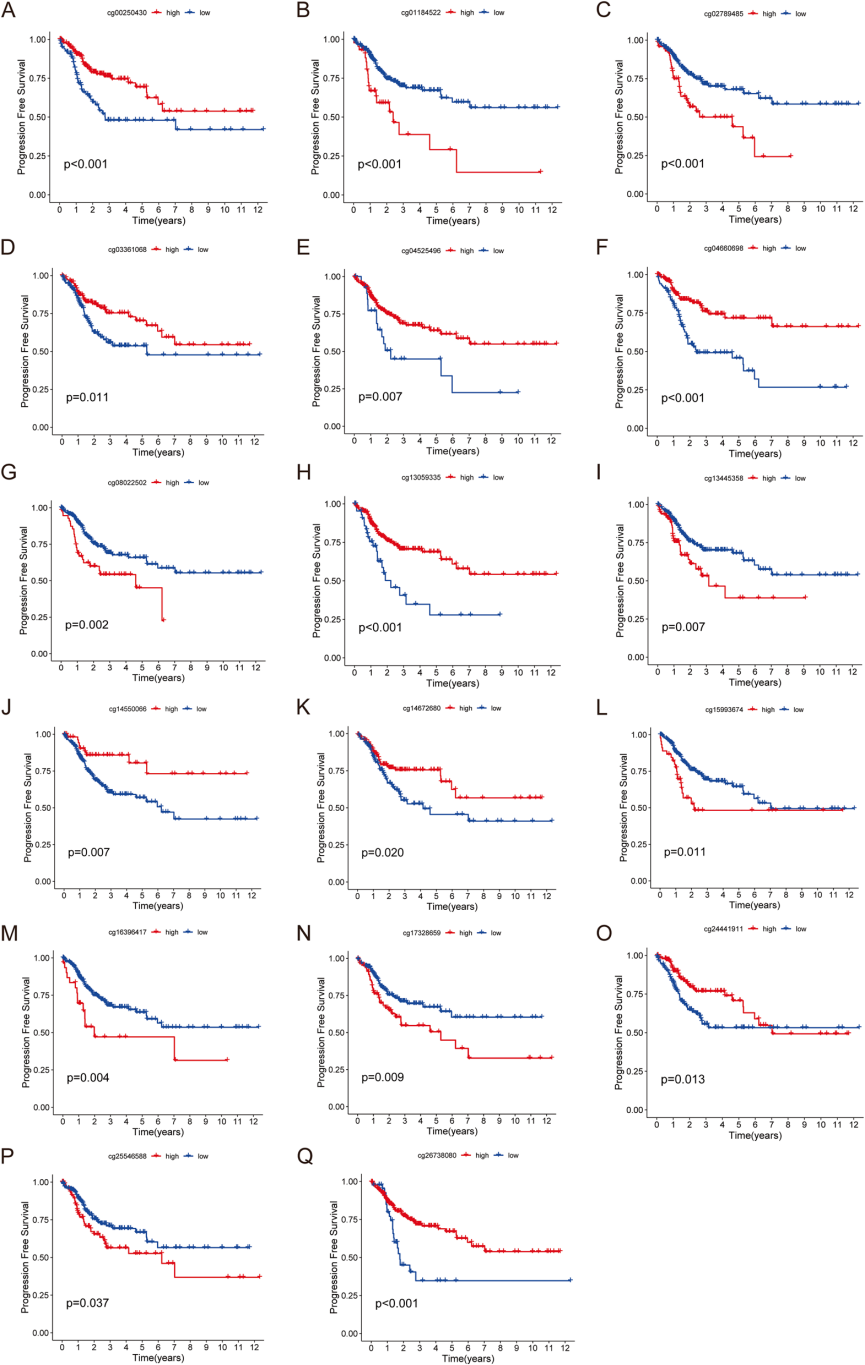
KM curve of 17-DMCs signature. **A**-**Q** cg00250430, cg01184522, cg02789485, cg03361068, cg04525496, cg04660698, cg08022502, cg13059335, cg13445358, cg14550066, cg14672680, cg15993674, cg16396417, cg17328659, cg24441911, cg25546588, and cg26738080

LASSO-Cox regression analysis identified methylation characteristics associated with PFS in CRC patients from 19 DMCs. 10-fold cross-validation was conducted for the model construction. When the number of methylation characteristics was 17, λ was the smallest, and the model was optimal (Fig. 5A-B). Therefore, these 17 DMCs as candidate methylation characteristics related to PFS in CRC patients. Based on the β values of these 17 DMCs and the corresponding regression coefficients, the following prognostic features were constructed: $\text{r}\text{i}\text{s}\text{k}\text{S}\text{c}\text{o}\text{r}\text{e}=\left(\text{c}\text{g}02789485\text{*}1.692\right)+(\text{c}\text{g}04660698\text{*}-32.388)+(\text{c}\text{g}00250430\text{*}-0.456)+\left(\text{c}\text{g}16,396,417\text{*}14.126\right)+(\text{c}\text{g}14,550,066\text{*}-0.655)+(\text{c}\text{g}24,441,911\text{*}-4.021)+(\text{c}\text{g}26,738,080\text{*}-0.403)+(\text{c}\text{g}04525496\text{*}-0.255)+(\text{c}\text{g}03361068\text{*}-0.834)+\left(\text{c}\text{g}01184522\text{*}1.356\right)+\left(\text{c}\text{g}25,546,588\text{*}5.063\right)+\left(\text{c}\text{g}17,328,659\text{*}0.839\right)+\left(\text{c}\text{g}13,445,358\text{*}6.063\right)+\left(\text{c}\text{g}15,993,674\text{*}0.709\right)+\left(\text{c}\text{g}08022502\text{*}0.242\right)+(\text{c}\text{g}14,672,680\text{*}-0.269)+(\text{c}\text{g}13,059,335\text{*}-1.596)$. CRC patients in the TCGA-COAD 450 K cohort (training dataset) and TCGA-COAD 27 K cohort (testing dataset) were divided into high-risk and low-risk groups based on the median risk score of methylation signatures. The KM analysis revealed that the PFS of patients in the high-risk group was significantly lower than that in the low-risk group in both the training dataset (Fig. 5C; Table 1) and the testing dataset (Fig. 5E). The ROC curve showed that the ROC AUC values of the prognostic model based on 17 DMCs for predicting 1-year and 3-year PFS of CRC patients in the training dataset were 0.754 and 0.785, respectively (Fig. 5D). The 1-year and 3-year ROC AUC values of the testing dataset were 0.651 and 0.658, respectively (Fig. 5F), which further verified the high accuracy of the prognostic model in predicting PFS of CRC patients. KM analysis showed that there was a significant difference in PFS between CRC patients in the high and low expression groups of 17DMCs (Fig. 6A-Q). All in all, the prognostic model based on 17 DMCs had good predictive value.

**Table 1:** Clinical information of TCGA-COAD 450 K datasets

Characteristics	High-risk	Low-risk
n	144	145
Gender, n (%)		
Female	65 (22.5%)	68 (23.5%)
Male	79 (27.3%)	77 (26.6%)
Stage, n (%)		
Stage I	16 (5.5%)	27 (9.3%)
Stage III	56 (19.4%)	28 (9.7%)
Stage II	40 (13.8%)	72 (24.9%)
Stage IV	27 (9.3%)	13 (4.5%)
Unknow	5 (1.7%)	5 (1.7%)
T, n (%)		
T2	17 (5.9%)	25 (8.7%)
T4	28 (9.7%)	10 (3.5%)
T3	95 (32.9%)	106 (36.7%)
T1	3 (1%)	4 (1.4%)
Unknow	1 (0.3%)	0 (0%)
M, n (%)		
M0	87 (30.1%)	110 (38.1%)
MX	27 (9.3%)	20 (6.9%)
M1	27 (9.3%)	13 (4.5%)
Unknow	3 (1%)	2 (0.7%)
N, n (%)		
N0	63 (21.8%)	105 (36.3%)
N1	50 (17.3%)	23 (8%)
N2	31 (10.7%)	17 (5.9%)

**Fig. 7:**
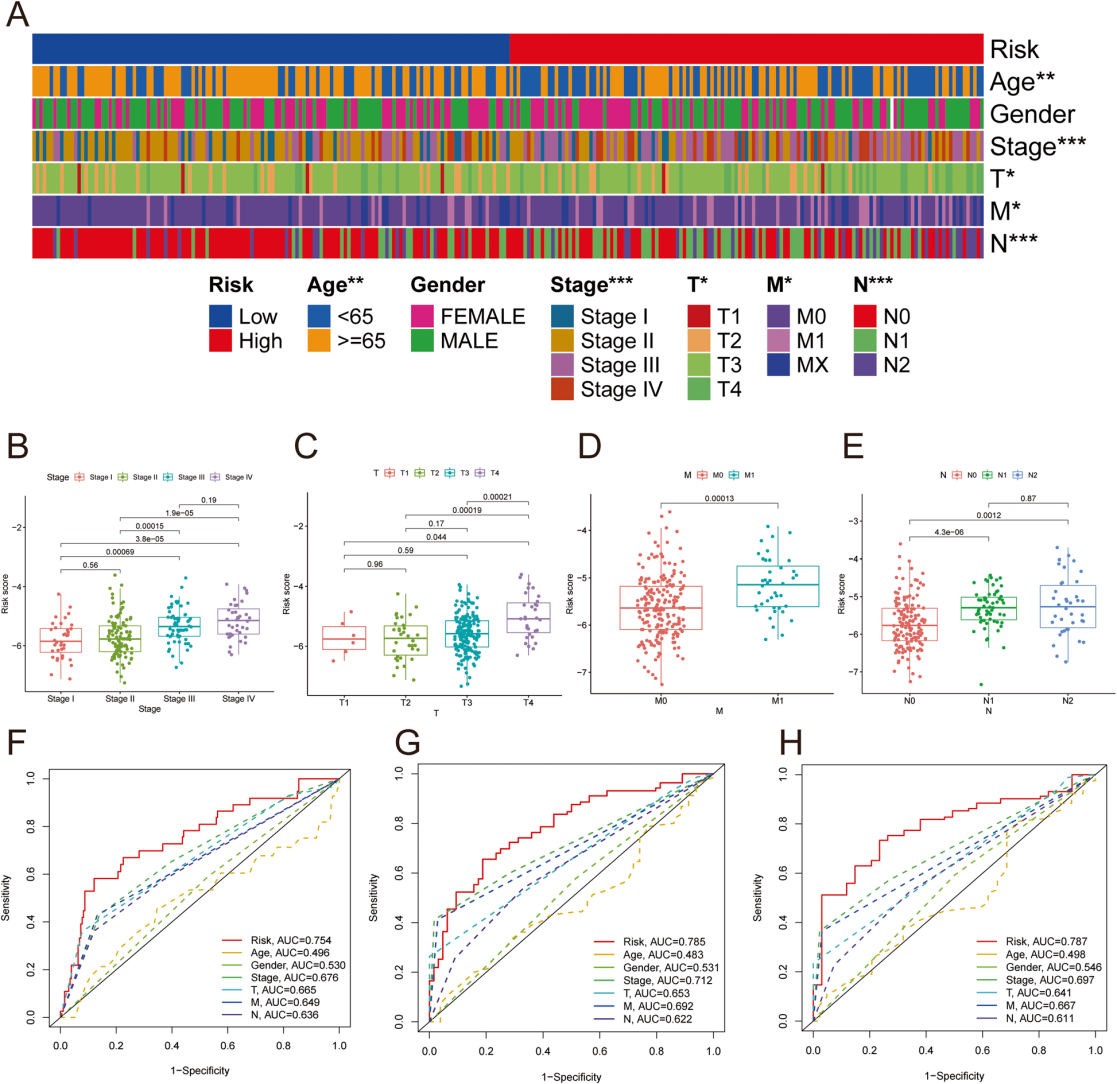
Correlation analysis between risk score and clinical indicators. **A** Heatmap for the connections between clinical indicators and the risk groups. The difference of risk score between different groups stratified by Age (**B**), Stage (**C**), T stage (**D**), and M stage (**E**). 1-year (**F**), 3-year (**G**) and 5-year (**H**) ROC curves of risk score and clinicopathological characteristics

Then, we explored the correlation between the prognostic models and pathological factors in the training dataset. In Fig. 7A, Age (*p* < 0.01), Stage (*p* < 0.001), T (*p* < 0.05), M (*p* < 0.05), and N (*p* < 0.001) were statistically different between the two risk subgroups. Among them, the Stage and N stages of CRC patients in the high-risk group were higher than those in the low-risk group. In addition, as the risk score increased, the Stage, T, M, and N stages also increased, implying that prognostic features may be associated with tumor enlargement and metastasis (Fig. 7B-D). In addition, we plotted the ROC curves of the prognostic model and clinicopathological features to predict the 1-, 3-, and 5-year PFS of CRC patients. The results showed that the prognostic model based on 17 DMCs had good prediction performance compared with clinicopathological features (Fig. 7F-H).

**Fig. 8:**
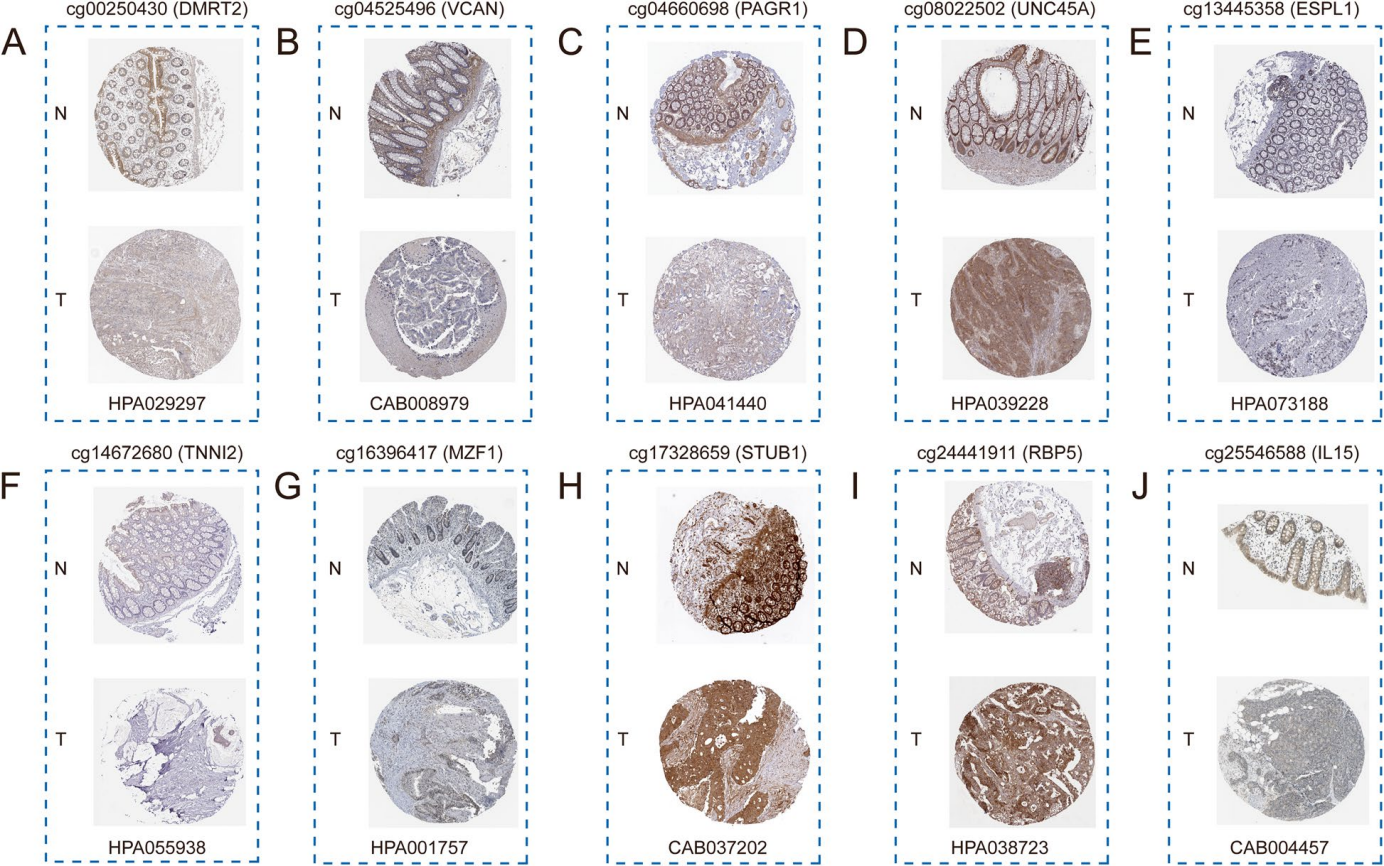
Immunohistochemistry analysis of genes where the prognostic DMCs was located. **A** cg00250430 (DMRT2). **B** cg04525496 (VCAN). **C** cg04660698 (PAGR1). **D** cg08022502 (UNC45A). **E** cg13445358 (ESPL1). **F** cg14672680 (TNNI2). **G** cg16396417 (MZF1). **H** cg17328659 (STUB1). **I** cg24441911 (RBP5). **J** cg25546588 (IL15). N represents normal cells, T represents tumor cells. Antibodies were marked below the box

Furthermore, we explored the expression at the protein level of genes in which prognostic related DMCs are located. From the HPA database, we searched for immunohistochemical maps of 10 genes associated with prognostic DMCs. Immunohistochemical analysis showed that the protein expression of the genes involved in prognostic DMCs was different between normal and diseased tissues. (Fig. 8A-J). Compared with normal tissues, PAGR1 (cg04660698) (staining: Low, intensity: Weak), VCAN (cg04525496) (staining: Low, intensity: Moderate), IL15 (cg25546588) (staining: Low, intensity: Weak), DMRT2 (cg00250430) (staining: Low, intensity: Weak) and STUB1 (cg17328659) (staining: Medium, intensity: Moderate) were lower in CRC, while MZF1 (cg16396417) (staining: Medium, intensity: Moderate), RBP5 (cg24441911) (staining: High, intensity: Strong), ESPL1 (cg13445358) (staining: High, intensity: Strong), UNC45A (cg08022502) (staining: High, intensity: Strong) and TNNI2 (cg14672680) (staining: Medium, intensity: Moderate) were highly expressed in CRC. Immunohistochemical information for prognostic genes can be found in Supplementary Material 5. In addition, we analyzed the methylation levels of genes (GLIPR1L2, PAGR1, MZF1, NCR1, RBP5, TNNC1, VCAN, TRIM9, ZNF496, IL15, DMRT2, STUB1, ESPL1, PRNP, UNC45A, TNNI2, ADAMDEC1) 17 prognostic related methylation sites. We input the above 17 genes into the Ualcan database (https://ualcan.path.uab.edu/) to obtain their methylation level changes across different stages (Stage I-IV). The results showed that the 17 genes mentioned above showed significant differences between different stages (Supplementary Material 6), indicating a close correlation between the methylation levels of the 17 genes and disease progression in CRC. This further confirmed that the 17 prognostic related methylation sites may play an important role in CRC by affecting gene transcription or expression.

The above results further confirmed that prognosis related DMCs may play an important role in the pathological progression of CRC.

### Nomogram model related to PFS of CRC

**Fig. 9:**
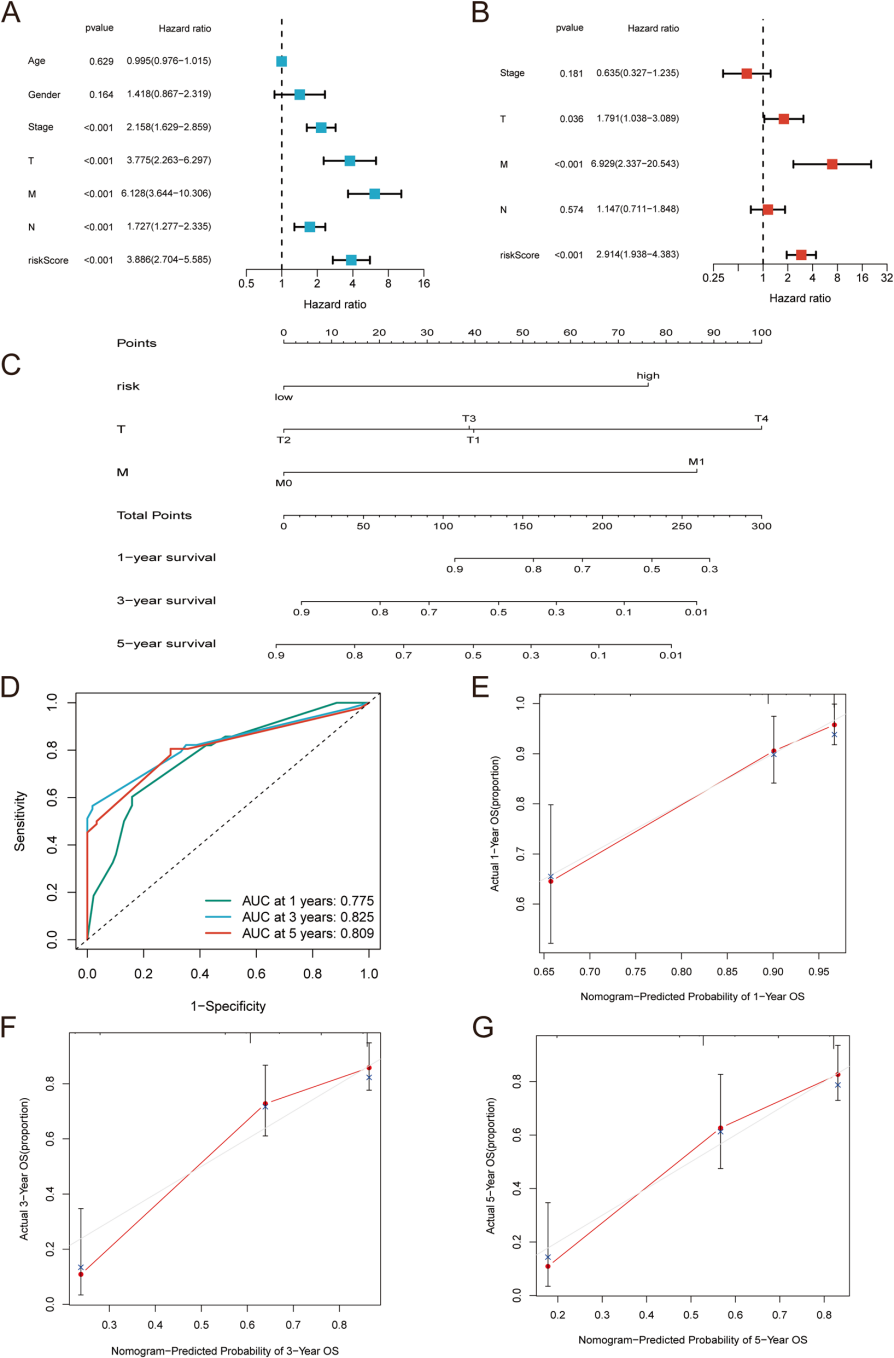
Construction of nomograph model. The forest plot of univariate Cox analysis (**A**) and multivariate Cox analysis (**B**). **C** Nomogram to predict the 1-year, 3-year, and 5-year PFS of CRC patients. **D** ROC curve to assess the predictive power of the nomogram model. 1-year (**E**), 3-year (**F**) and 5-year (**G**) calibration curves of nomograph models

Univariate Cox and multivariate Cox regression analyses were performed on risk score and clinical characteristics in the training dataset to screen features that could independently predict PFS in CRC. Figure 9A and B (Supplementary Material 7) show that risk score, T, and M were independent predictors of PFS for CRC. Then, we constructed a nomogram model based on the risk score, T and M, to predict the PFS of CRC patients at 1-, 3-, and 5 years (Fig. 9C). To estimate the predictive performance of the nomogram, we plotted the ROC curve and calibration curve of the nomogram model. The ROC curve revealed that the nomogram model had high accuracy in predicting the PFS of CRC patients at 1 year (AUC = 0.775), 3 years (AUC = 0.825), and 5 years (AUC = 0.809) (Fig. 9D). In addition, the calibration curve showed that the prediction results of the nomogram model at 1 year and 5 years were in good agreement with the actual observed values (Fig. 9E-G).

### Immune differences between risk subgroups

**Fig. 10:**
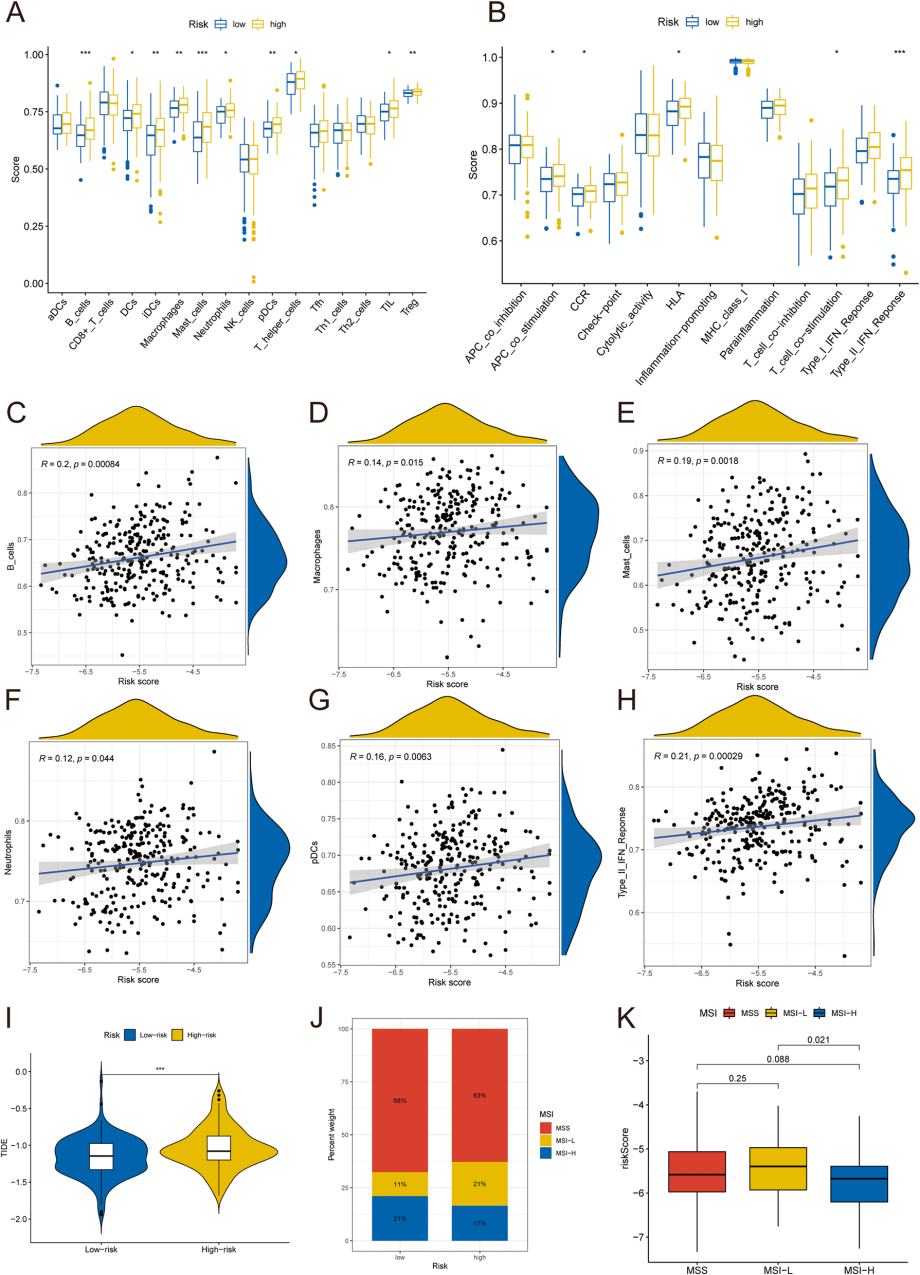
Comparison of immune microenvironment between two risk subgroups. Differences in the abundance of immune cells (**A**) and immune function (**B**) among risk subgroups. **C**-**H** Correlation between risk score and B_cells, Macrophages, Mast_cells, Neutrophils, pDCs, Type_II_IFN_Reponse respectively. **I** Differences in TIDE scores between high-risk and low-risk groups. **J** The proportion of MSS and MSI (MSI-H and MSI-L) in risk subgroups. **K** The difference of risk score among MSS subtype, MSI-H subtype and MSI-L subtype

According to the results of ssGSEA, we found that the scores of immune cells and immune function were generally higher in the high-risk group than in the low-risk group. Figure 10A showed that the scores of B_cells, DCs (Dendritic Cells), iDCs (Immature Dendritic Cells), Macrophages, Mast_cells, Neutrophils, pDCs (Plasmacytoid Dendritic Cells), T_helper_cells, Tumor Infiltrating Lymphocytes (TIL) and Regulatory T cells (Treg) were significantly different between the high-risk and low-risk groups. Figure 10B showed that the scores of APC_co_stimulation, CCR, HLA, T_cell_co-stimulation, and Type_II_IFN_Reponse were significantly different between the two risk subgroups. The risk score was highly correlated with B_cells, Macrophages, Mast_cells, Neutrophils, pDCs, and Type_II_IFN_Reponse. Next, we performed immune correlation analysis on DNA methylation data in the TCGA-COAD cohort using HiTIMED. The results showed that there was a significant difference in the immune scores of CD8nv and DC between the risk groups (Supplementary Material 8). Moreover, as the risk score increased, so did the score for immune cells or pathways (Fig. 10C-H). TIDE score can be used to screen patients suitable for immunotherapy. The TIDE score, the proportion of MSI, and the proportion of MSI-L in the high-risk group were significantly higher than those in the low-risk group (Fig. 10I-J). In addition, with the increase of risk score, MSI-L was significantly higher than MSI-H (Fig. 10K, *p* = 0.021). These results suggested that the prognosis of patients in the high-risk group and the response to immunotherapy are worse, and immunotherapy helps to improve the prognosis of patients in the low-risk group.

### Differences in TMB levels between risk subgroups

**Fig. 11:**
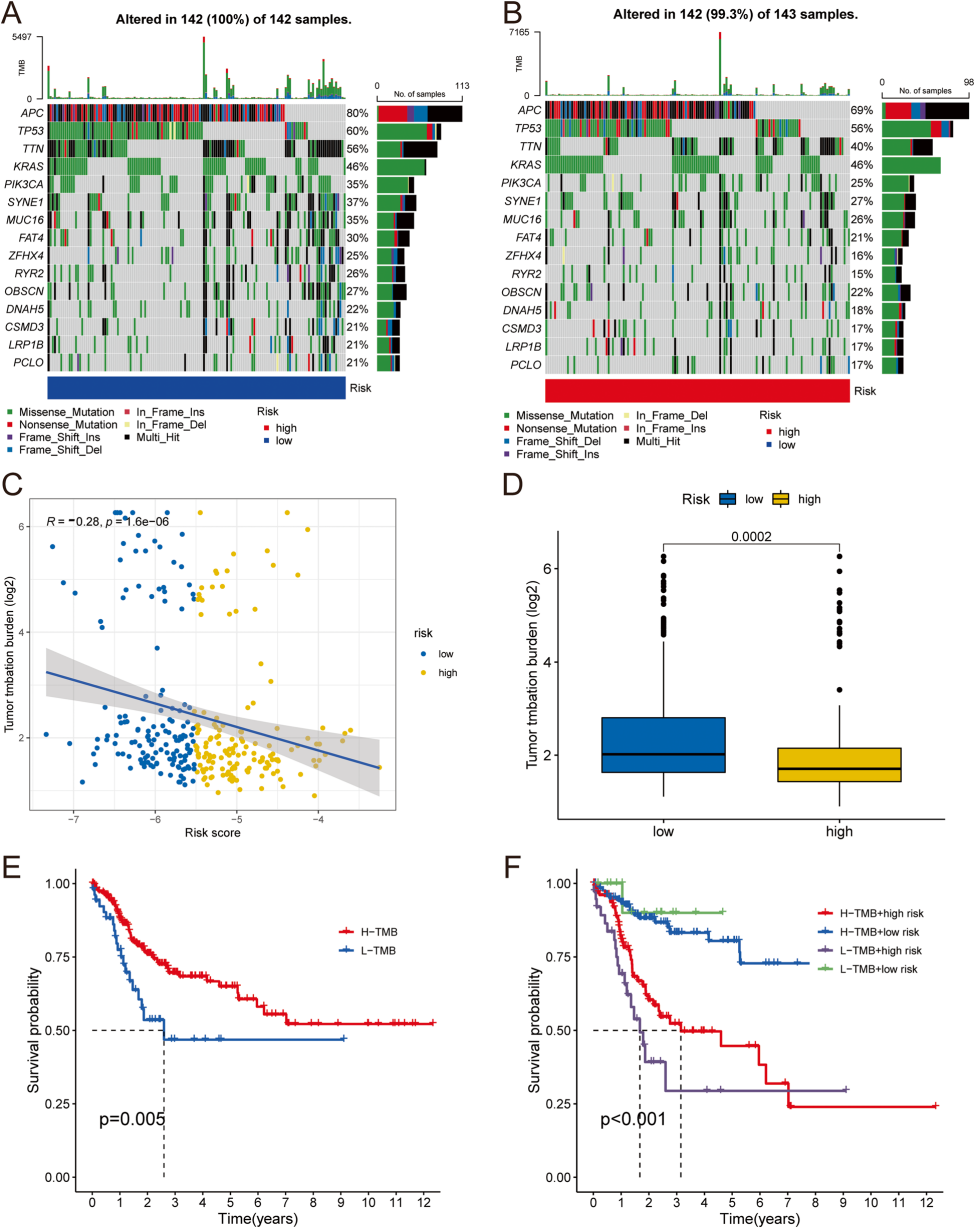
Landscape of TMB between risk subgroups. Waterfall showing the top 20 mutated genes in low-(**A**) and high-risk group (**B**). **C** Correlation between risk score and TMB level. **D** Differences in TMB levels between risk subgroups. **E** Survival curve showing different PFS between high- and low-TMB groups in training dataset. **F** Survival curve of the comprehensive analyses between TMB and risk score

TMB levels are highly correlated with the prognosis of cancer patients. Therefore, we investigated the TMB differences between the high- and low-risk groups in the training dataset. Figure 11A and B revealed that the mutation rate of patients in the high-risk group was slightly lower than that in the low-risk group. Risk scores were negatively correlated with TMB levels (Fig. 11C), and the TMB level (log2) of the low-risk group was significantly higher than that of the high-risk group (*p* = 0.0002, Fig. 11D). KM analysis results revealed that patients in the H-TMB group had higher PFS than those in L-TMB (Fig. 11E), and patients with low TMB in the high-risk group had the worst PFS (Fig. 11F). Studies have found that patients with high TMB levels respond better to immunotherapy, suggesting that immunotherapy is less effective in improving outcomes in CRC patients. These results further confirmed that patients in the high-risk group have a poorer prognosis and sensitivity to immunotherapy than those in the low-risk group, and patients in the low-risk group are more suitable for immunotherapy.

## Discussion

Abnormal DNA methylation can not only be used as a target for cancer therapy but also for disease diagnosis and prognosis prediction [14, 15]. In addition, changes in DNA methylation mainly occur in the early stage of carcinogenesis and cancer progression, which makes DNA methylation characteristics conducive to the early prevention and diagnosis of disease. Metastasis and recurrence are the key factors affecting the treatment and survival of CRC patients. DNA methylation features have great potential as biomarkers related to CRC progression and prognosis. Therefore, our study aimed to elucidate the landscape of DNA methylation during CRC metastasis and assess its clinical guiding value in CRC.

Firstly, we identified DMCs between CRC patients with and without metastasis. Most DMCs were found to have increased β values in CRC patients with metastasis compared to CRC patients without metastasis. It has been reported that many tumor suppressor genes are partially or wholly silenced due to hypermethylation of their CpG sites [16], suggesting that the hypermethylation level of CpG sites may play a vital role in the occurrence and progression of CRC metastasis. The results of enrichment analysis showed that the genes where DMCs are located were mainly enriched in Neuroactive ligand-receptor interaction, cAMP signaling pathway, transcription by RNA polymerase II, cognition, and cell-cell adhesion. In the process of cancer BMS, Neuroactive ligand-receptor interaction plays a prominent role in adapting to the environment of target organs [17]. cAMP signaling pathway can regulate the ability of colon cancer metastasis [18]. Guo et al. found that Tetraspanin CO-029 participated in cancer metastasis in the digestive system by regulating cell-cell adhesion [19]. The biological pathways obtained from the enrichment of genes where DMCs are located related to the progression of CRC, suggesting that DMCs may also play an important role in CRC.

In order to evaluate the prognostic ability of DMCs, univariate Cox regression analysis and KM test were performed for DMCs, and a total of 20 candidate DMCs related to PFS in CRC patients were screened. Based on the PFS-related candidate DMCs, 19 DMCs were selected by the SVM-RFE algorithm for the construction of the diagnostic model and nomogram model. ROC analysis showed that the 19-DMCs diagnostic model could predict the metastasis of CRC patients with high accuracy. The calibration curve, DCA analysis, and clinical impact curve showed that the 19-DMCs nomogram model had good clinical guiding value in predicting CRC metastasis. These results indicated that 19 DMCs may play a key role in CRC metastasis. Based on 19 DMCs, LASSO-Cox regression analysis was utilized to construct 17 DMCs (cg02789485, cg04660698, cg00250430, cg16396417, cg14550066, cg24441911, cg26738080, cg04525496, cg03361068, cg01184522, cg25546588, cg17328659, cg13445358, cg15993674, cg08022502, cg14672680, and cg13059335). The ROC curve showed that the prognostic model could predict 1-year and 3-year PFS of CRC patients with medium-high accuracy. Compared with the actual observed values, the nomogram model can better predict the 1-year and 5-year PFS of CRC patients.

The alteration of CpG sites has an important effect on gene expression. We annotated 17 prognostic DMCs genes: cg02789485 (GLIPR1L2), cg04660698 (PAGR1), cg16396417 (MZF1), cg14550066 (NCR1), cg24441911 (RBP5), cg26738080 (TNNC1), cg04525496 (VCAN), cg03361068 (TRIM9), cg01184522 (ZNF496), cg25546588 (IL15), cg00250430 (DMRT2), cg17328659 (STUB1), cg13445358 (ESPL1), cg15993674 (PRNP), cg08022502 (UNC45A), cg14672680 (TNNI2), cg13059335 (ADAMDEC1). GLIPR1L2 is a novel target gene of tumor suppressor gene p53, and p53 mutations occur in 60% of CRC [20, 21]. PAGR1 can affect fat generation by regulating C/ EBp-β and C/ EBp-δ [22]. Multiple studies have shown that fat is one of the risk factors for CRC [23]. Studies have found that MZF1 can promote the proliferation of tumor cells in CRC and inhibit cancer progression through apoptosis [24]. In mouse models, NCR1-mediated IFN-γ production leads to increased expression of FN1 in tumors, thereby altering the primary tumor structure and reducing tumor metastasis [25]. Wan et al. found that deletion of RBP5-mediated protein delayed tumor progression in a mouse model of cholangiocarcinoma [26]. Studies have shown that TNNC1 is a promising biomarker for metastasis of ovarian and tongue cancers [27, 28]. The upregulation of VCAN mediated by INHBA can promote the migration and proliferation of cancer cells in CRC [29]. Cui et al. found that IFN-γ produced by NK cells could affect the proliferation of CRC cells through the regulation of IL-15 [30]. In some CRC samples, the proportion of CHIP (aka STUB1) increased and was associated with poor survival [31]. Studies have found that, compared with normal samples, PRNP expression is up-regulated in CRC and is an independent prognostic factor for 3-year survival of CRC [32]. A large amount of evidence has revealed that the expression of UNC45A in cancer cells is related to the proliferation and metastasis of solid tumors [33]. Through comprehensive biogenic analysis, Li et al. found that TNNI2 was identified as a prognostic biomarker for CRC. In addition, the model based on 7 genes including TNNI2 can predict CRC metastasis (liver or lung), with AUC of 0.933 [34]. Macartney-Coxson et al. identified ADAMDEC1 as a candidate gene associated with CRC liver metastasis, whose mRNA and protein expression decreased during the occurrence and progression of CRC [35]. In summary, 17 prognostic-related DMCs genes play an important role in the occurrence and progression of CRC, which further verifies that 17 prognostic-related DMCs may also play a key role in CRC.

DNA methylation is essential for the interaction between tumors and immune cells [36]. In this study, we found that the scores of immune cells and immune-related pathways in the high-risk group were generally higher than those in the low-risk group. Multiple immune cells or immune-related pathways, such as Macrophages, Mast_cells, pDCs, and Type_II_IFN_Reponse, have significant differences between the two risk subgroups. In addition, as the risk score increased, so did the score of immune cells or immune-related pathways. The interaction between the extracellular trap of macrophages and colon cancer cells promotes CRC invasion [37]. Neutrophils and their releases have been associated with the progression and metastasis of various cancers, and targeting neutrophils in extracellular traps may be an effective strategy to inhibit the metastasis of colorectal cancer [38]. Type_II_IFN_Reponse is involved in cancer’s immune response regulation mechanism and is related to the growth and migration ability of cancer cells [39]. We also found that TIDE scores were higher in high-risk patients than in low-risk patients. Furthermore, patients with high TMB had higher PFS than those with low TMB, and TMB levels decreased in CRC patients as risk scores increased. These results indicated that the low-risk patients had better immunotherapy response.

There are some limitations to this study. We need more CRC 450 K DNA methylation array data to validate the DNA methylation-related prognostic model. In addition, the treatment methods of CRC patients have some potential impacts on their prognosis, including whether the treatment methods are effective for patients, the interaction effects between multiple treatment methods, genetic variations of patients, and their sensitivity to treatment methods. Therefore, there is an urgent need for more precise personalized treatment to help patients choose more effective treatment methods and effectively improve their prognosis.

## Conclusion

In short, we identified DNA methylation biomarkers associated with CRC metastasis and constructed a 19 DMCs correlation diagnostic model and a line graph model that can accurately predict CRC metastasis. We also identified DNA methylation biomarkers associated with PFS in CRC patients and constructed a 17 DMCS-related prognostic model that predicted PFS in CRC patients with moderate to high accuracy. In addition, we elucidate the association of TME, MSI, and TMB with DNA methylation-related prognostic models. Our results may provide new biomarkers for predicting metastasis in CRC patients and PFS.

### Supplementary Information

Supplementary Material 1.

Supplementary Material 2.

Supplementary Material 3.

Supplementary Material 4.

Supplementary Material 5.

Supplementary Material 6.

Supplementary Material 7.

Supplementary Material 8.

## Data Availability

All data can be obtained in TCGA and GEO databases.
