# Supplementary figures and images for: Identification of DNA methylation characteristics associated with metastasis and prognosis in colorectal cancer
Source: BMC Med Genomics. 2024 May 10;17:127. doi: 10.1186/s12920-024-01898-4 (PMC11088068; doi:10.1186/s12920-024-01898-4)

1. cg04660698 (PAGR1)


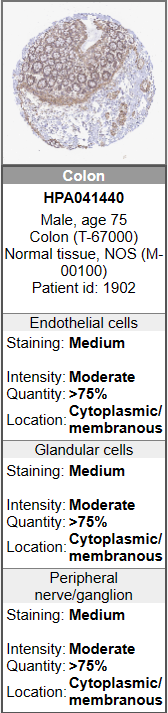

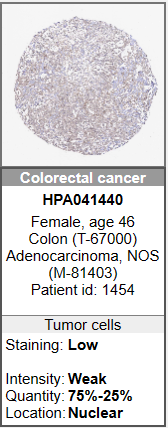


2. cg16396417 (MZF1)


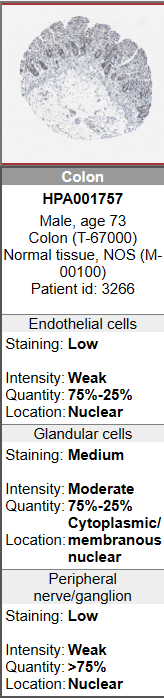

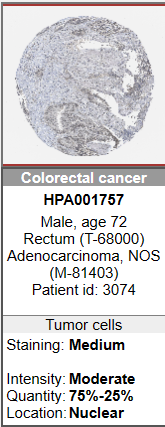


3. cg24441911 (RBP5)


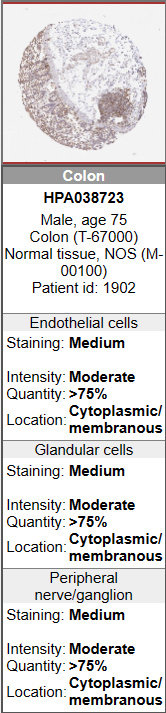

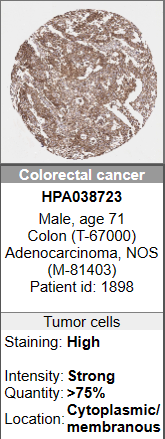


4. cg04525496 (VCAN)


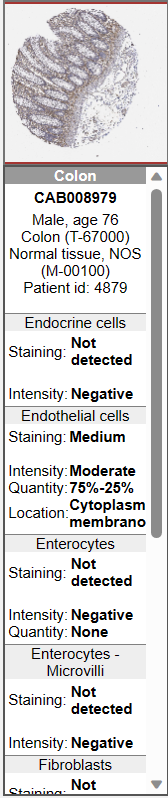

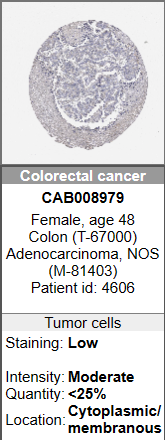


5. cg25546588 (IL15)


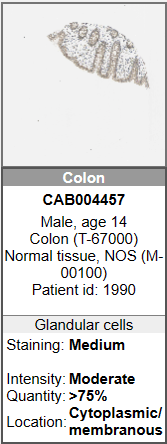

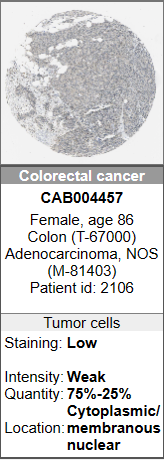


6. cg00250430 (DMRT2)


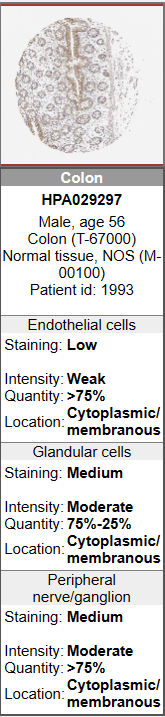

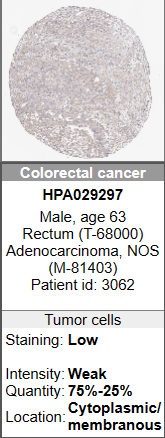


7. cg17328659 (STUB1)


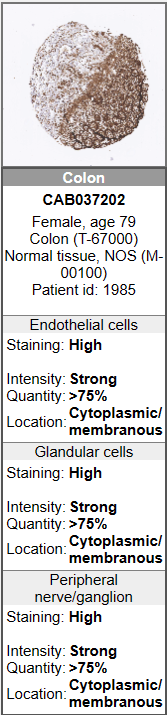

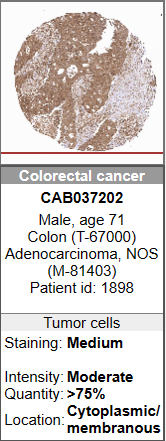


8. cg13445358 (ESPL1)


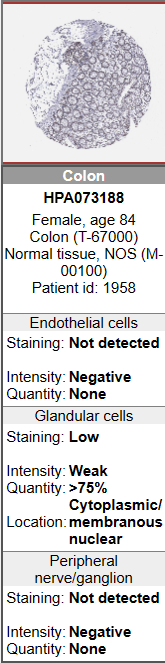

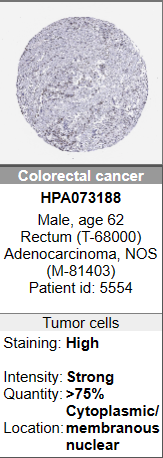


9. cg08022502 (UNC45A)


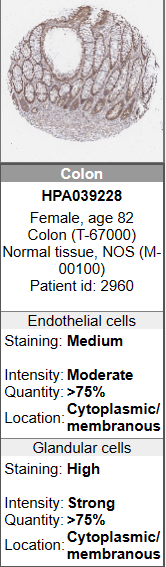

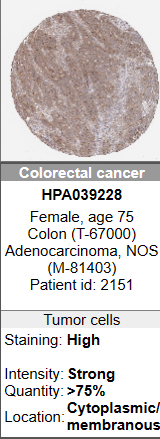


10. cg14672680 (TNNI2)


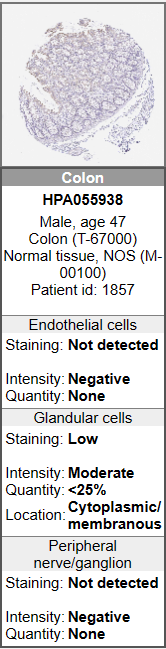

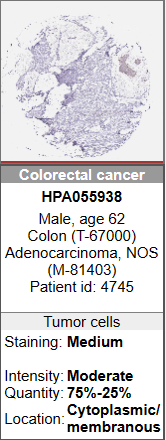

Supplement: Supplementary file 5 — Supplementary Material 5. [file 12920_2024_1898_MOESM5_ESM.docx]

GLIPR1L2


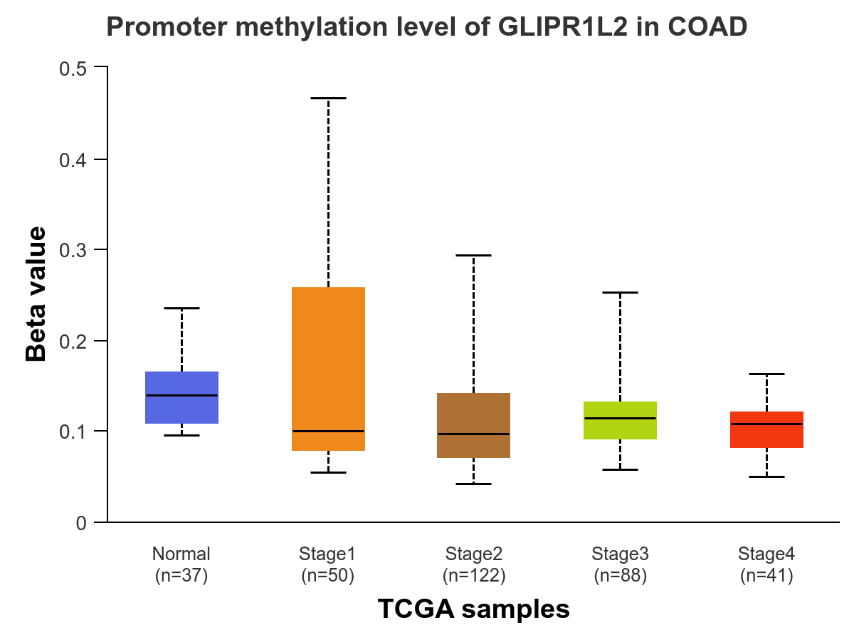

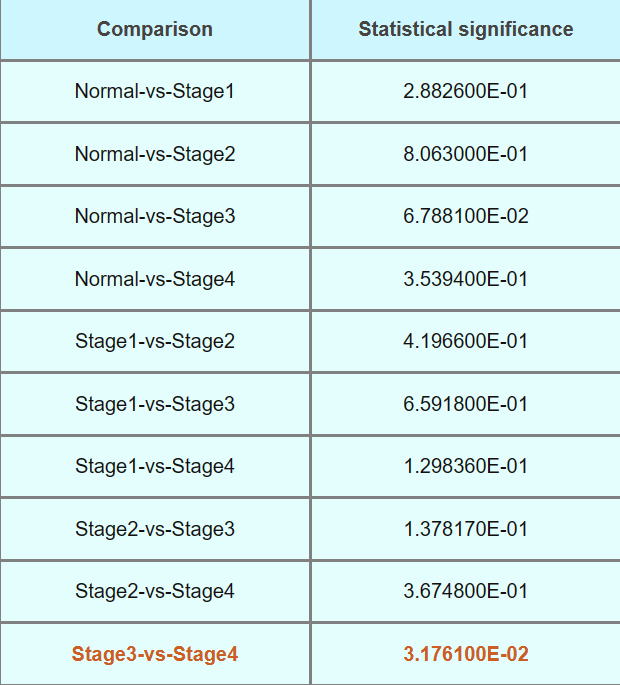


MZF1


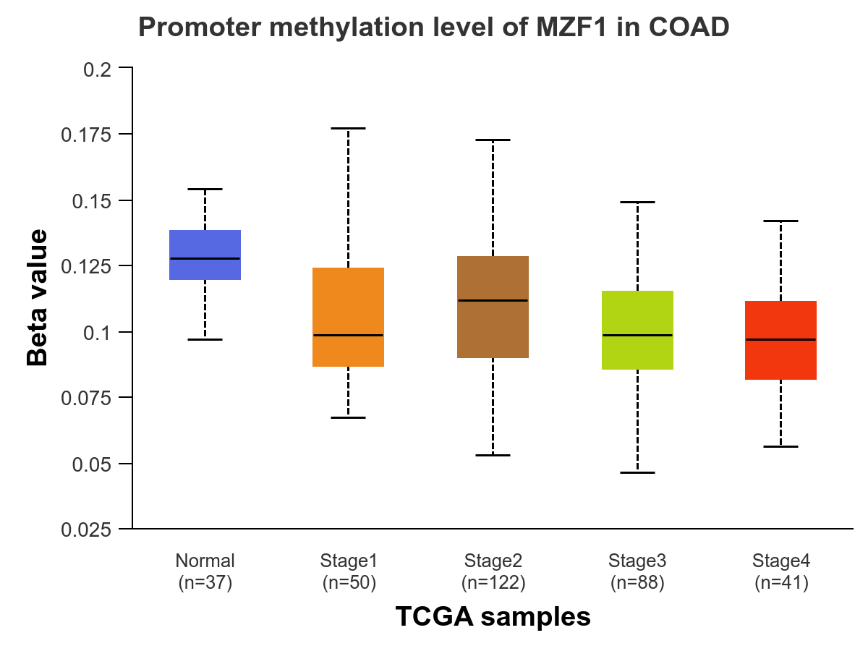

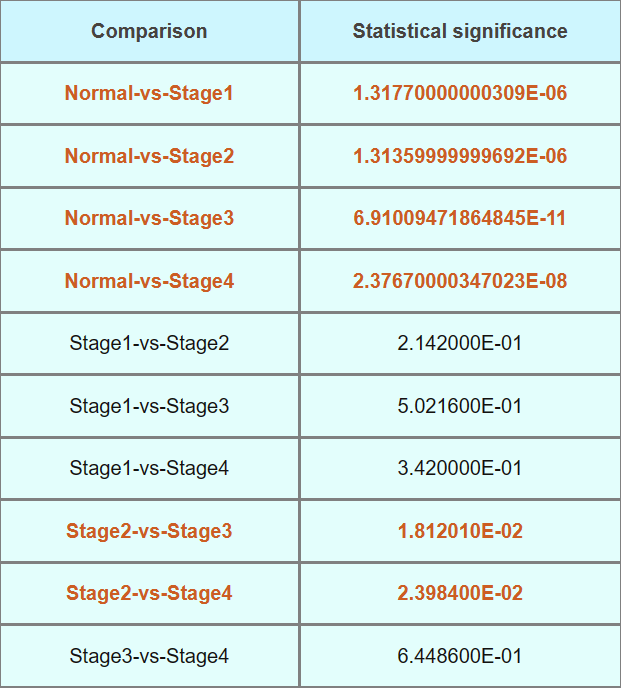


NCR1


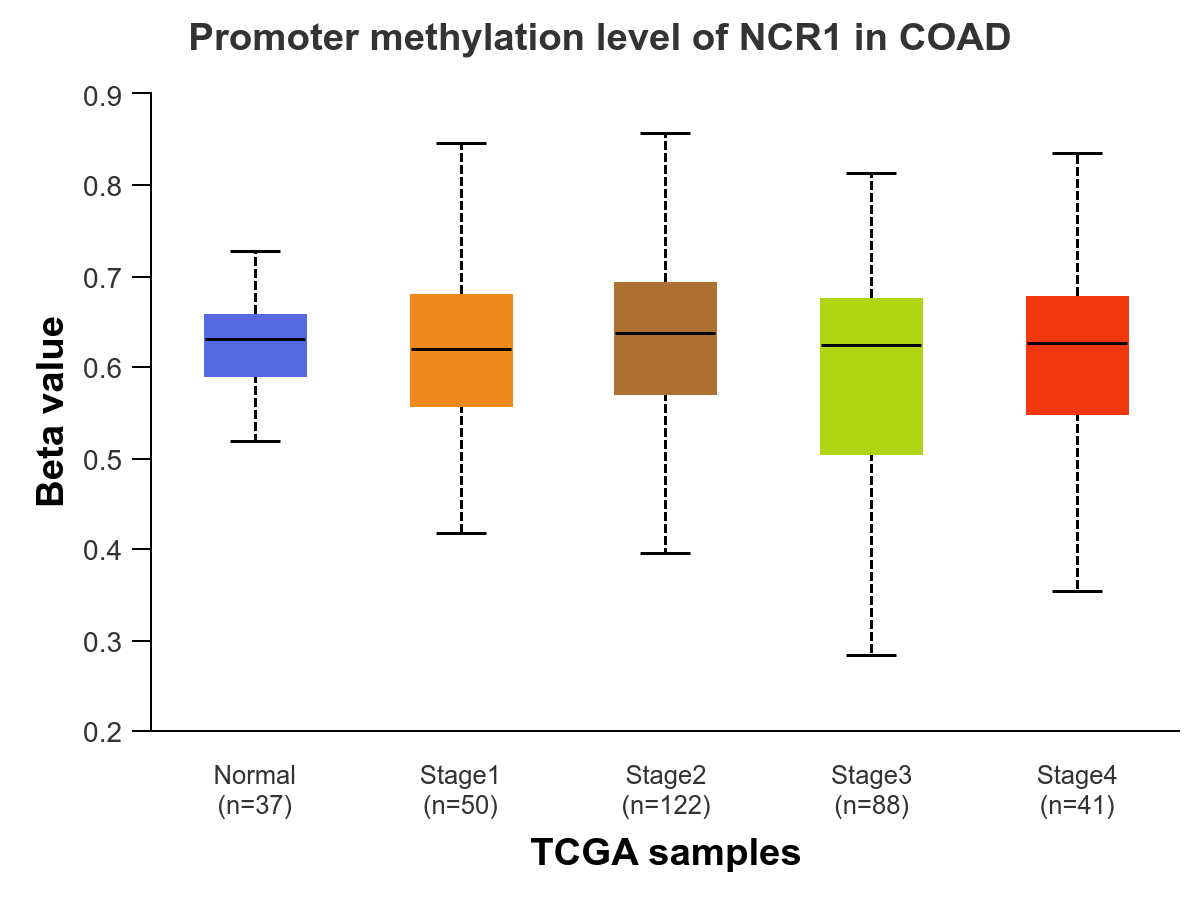


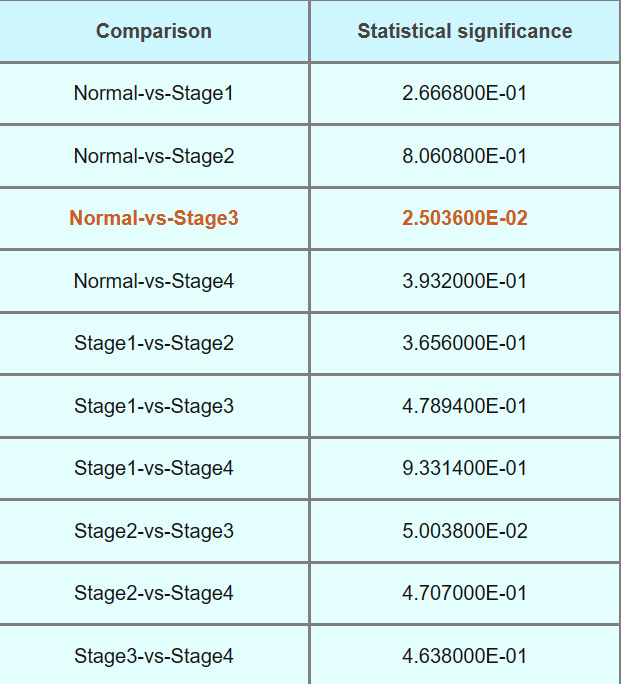


RBP5


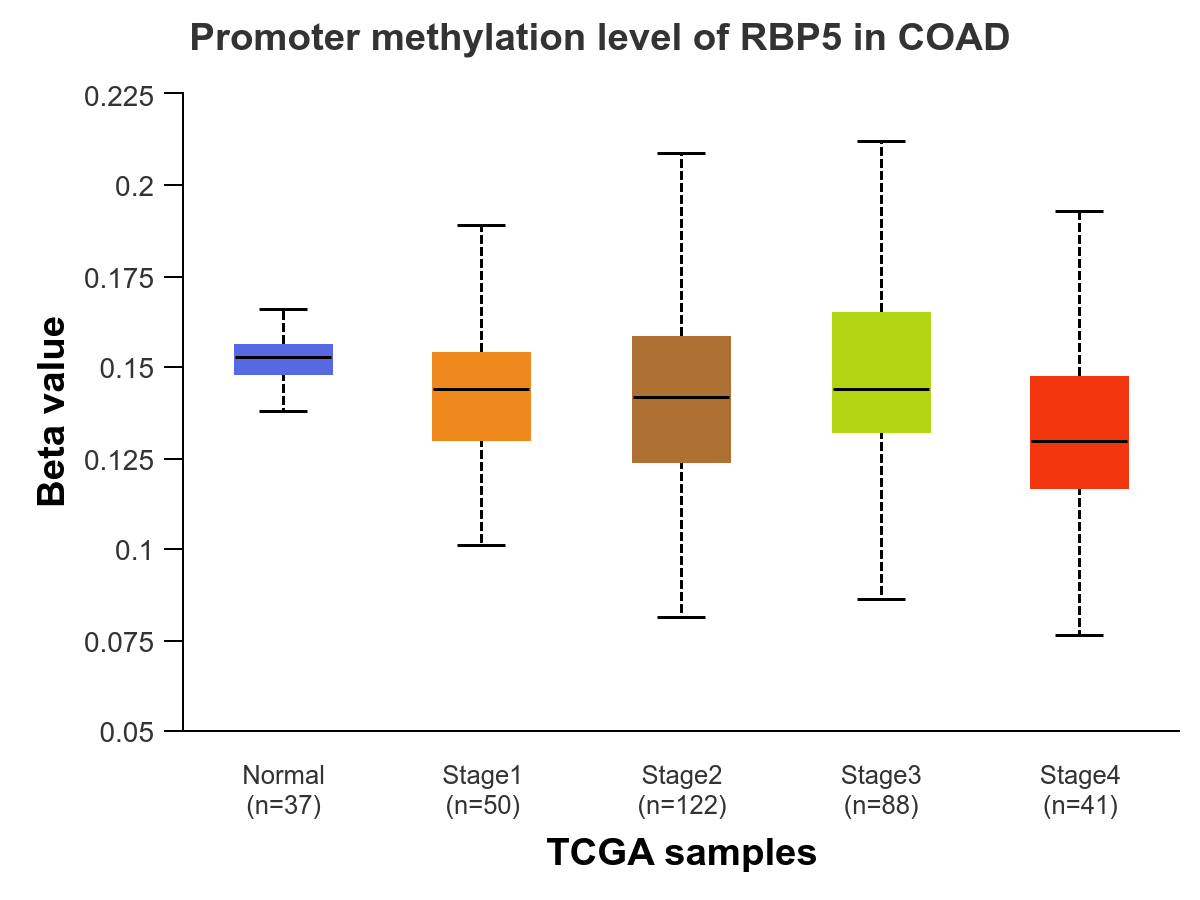


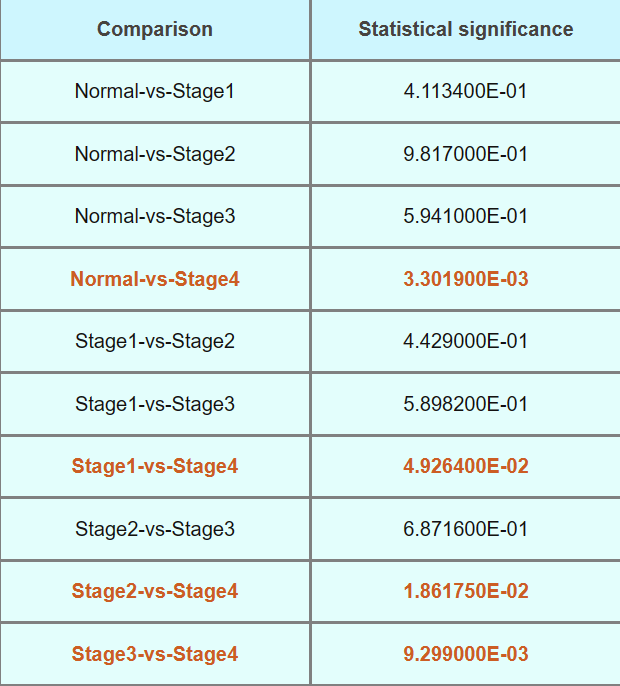


TNNC1


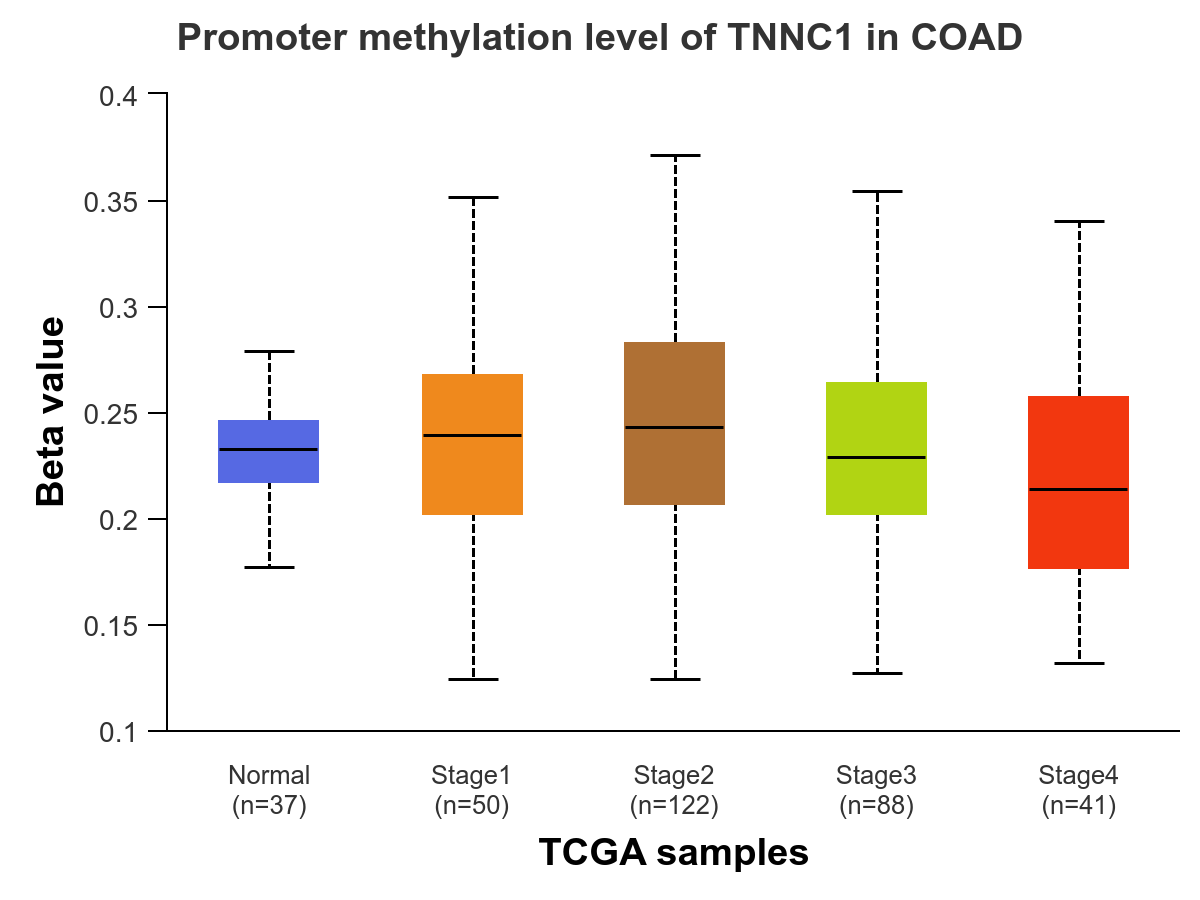


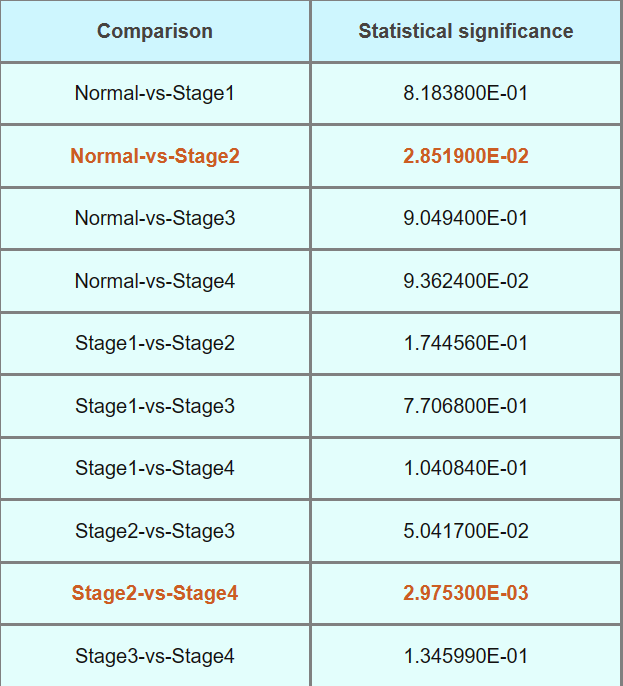


VCAN


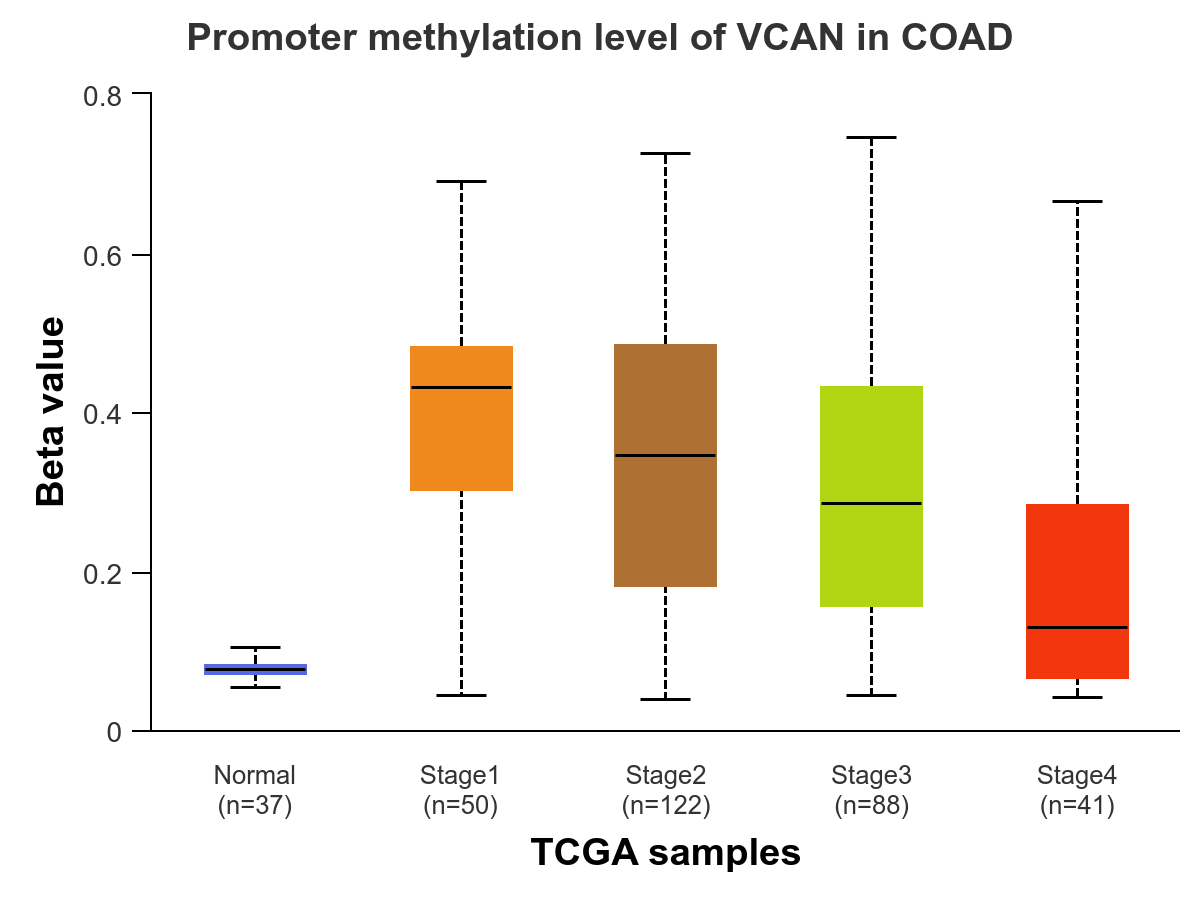


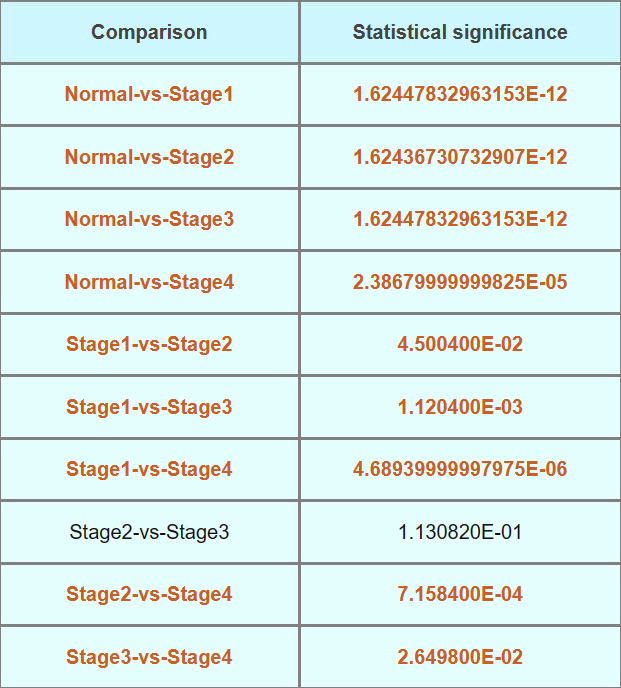


TRIM9


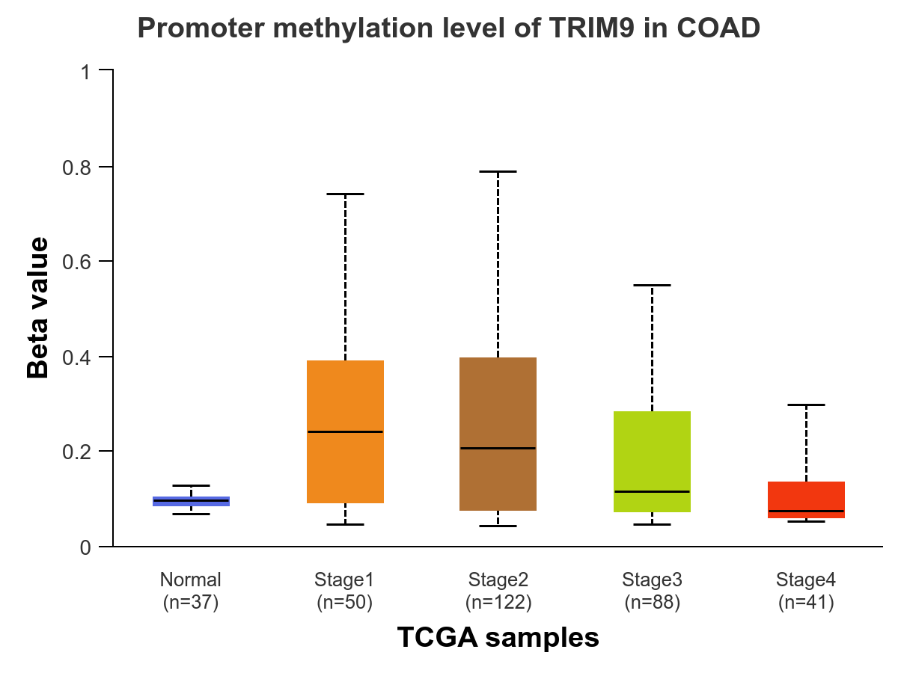


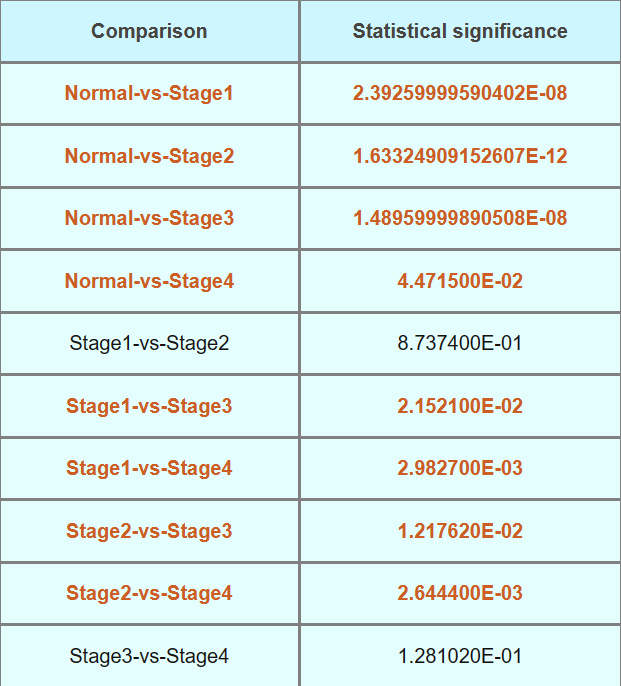


ZNF496


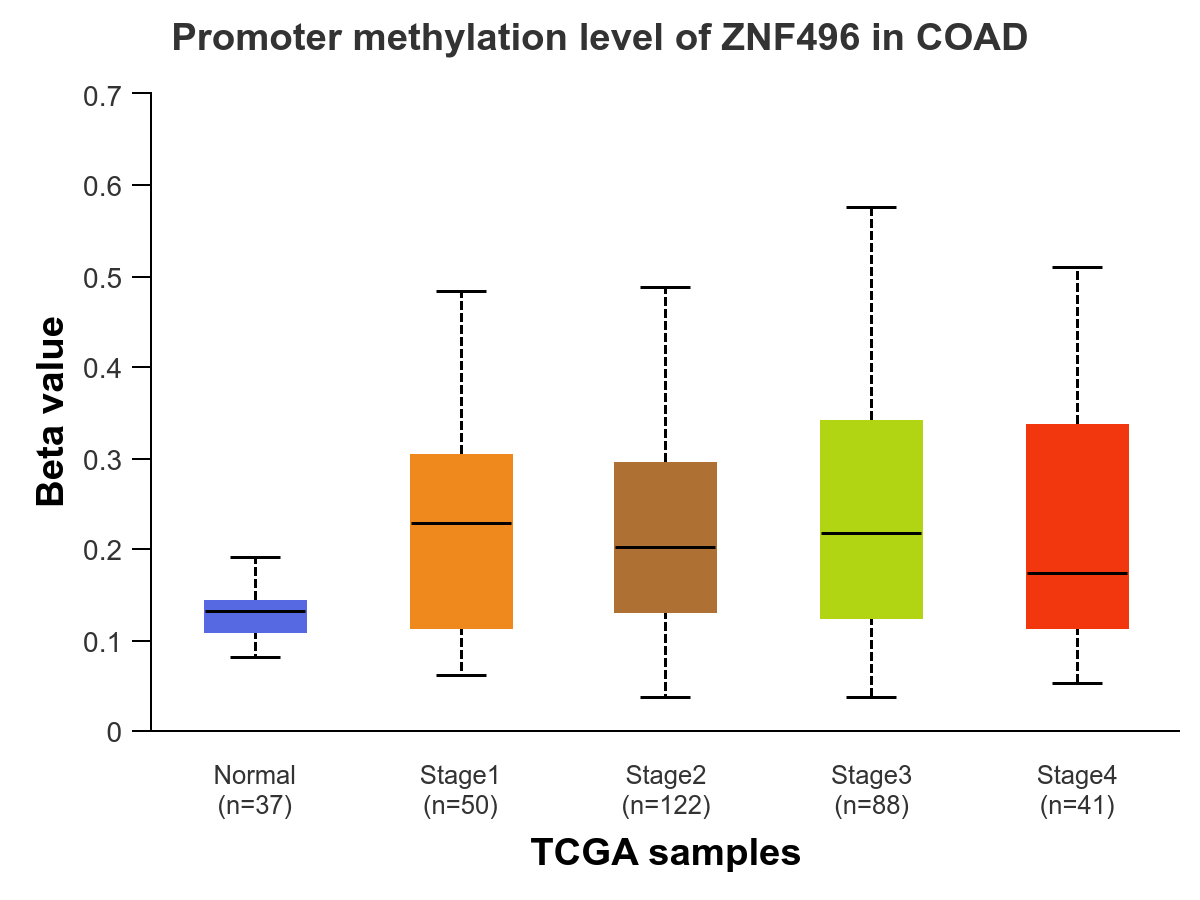


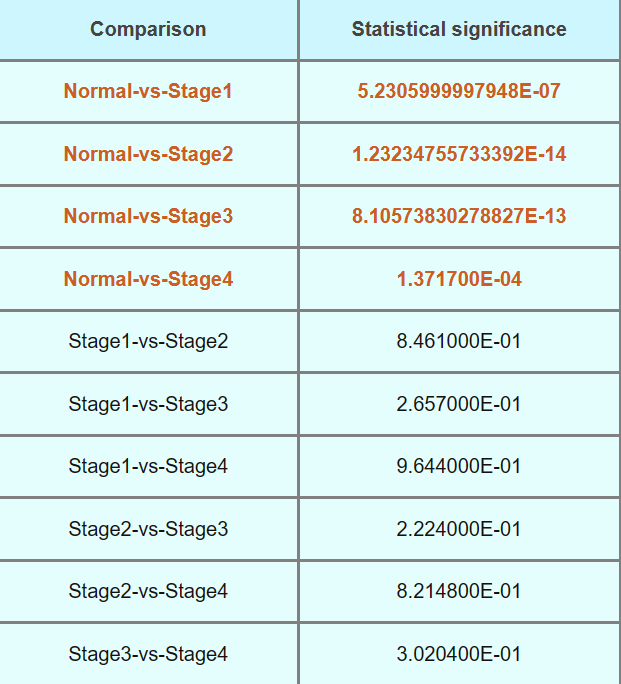


IL15


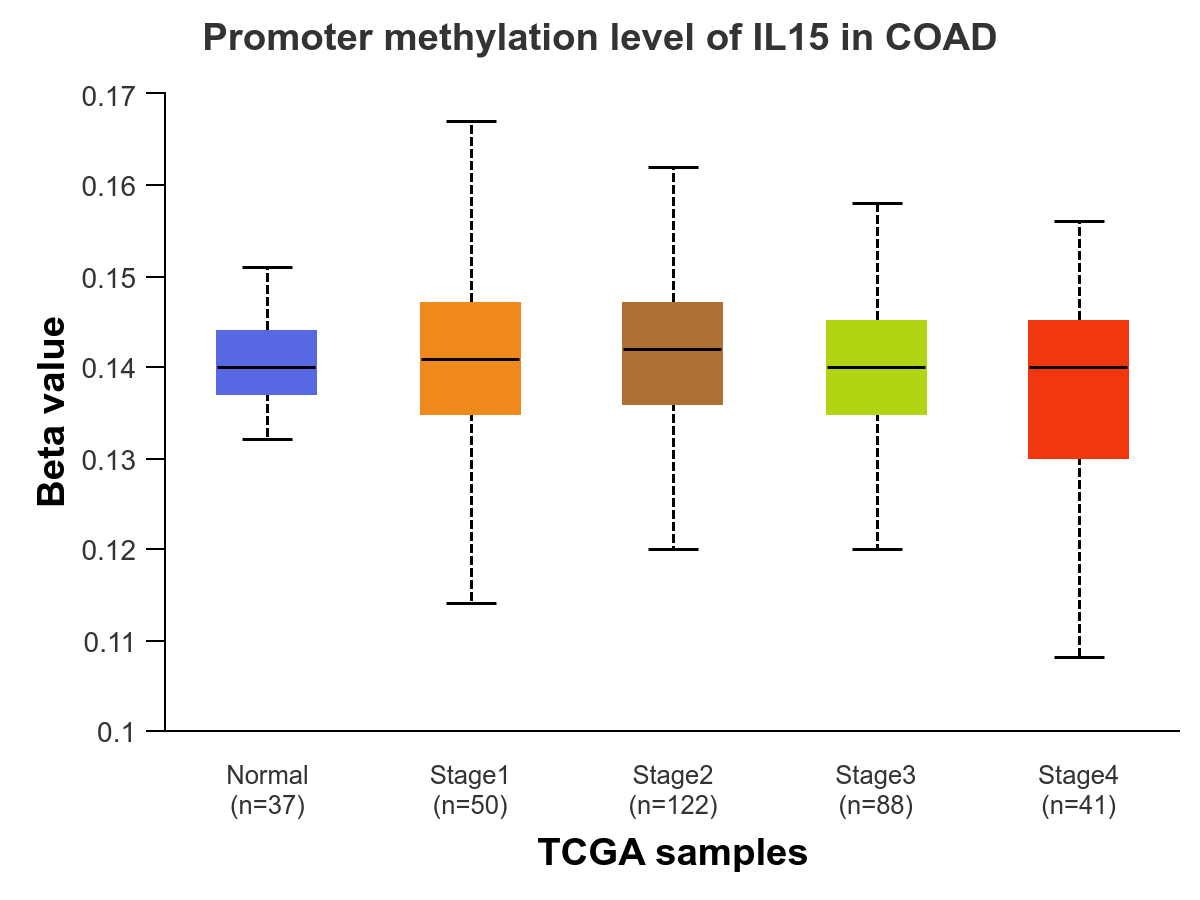


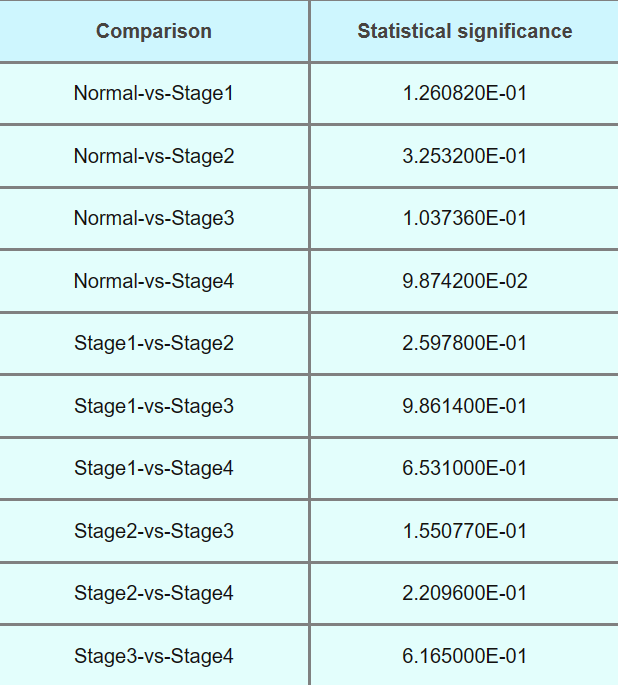


DMRT2


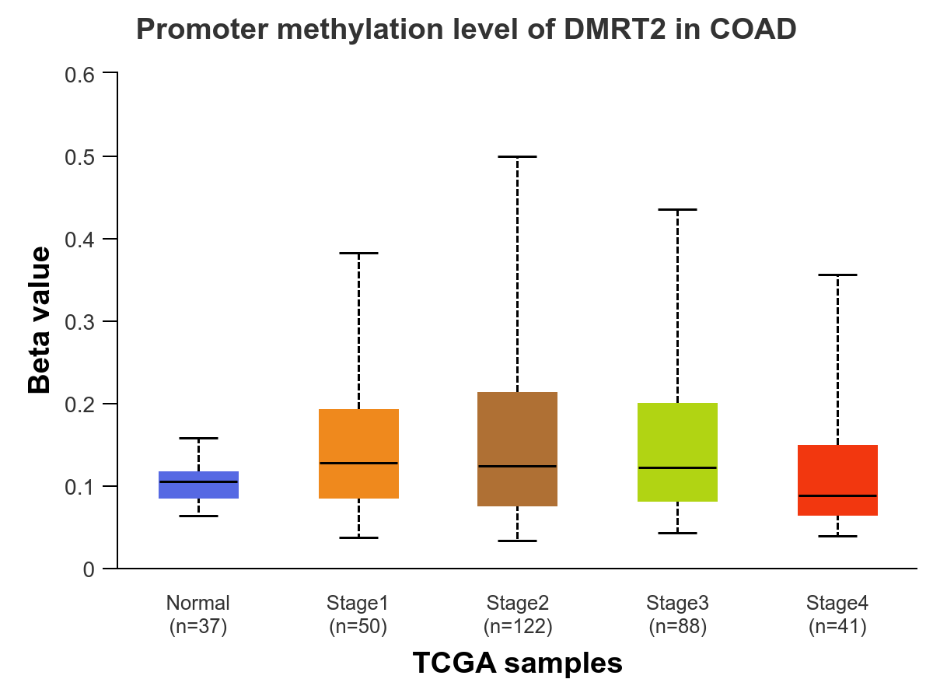


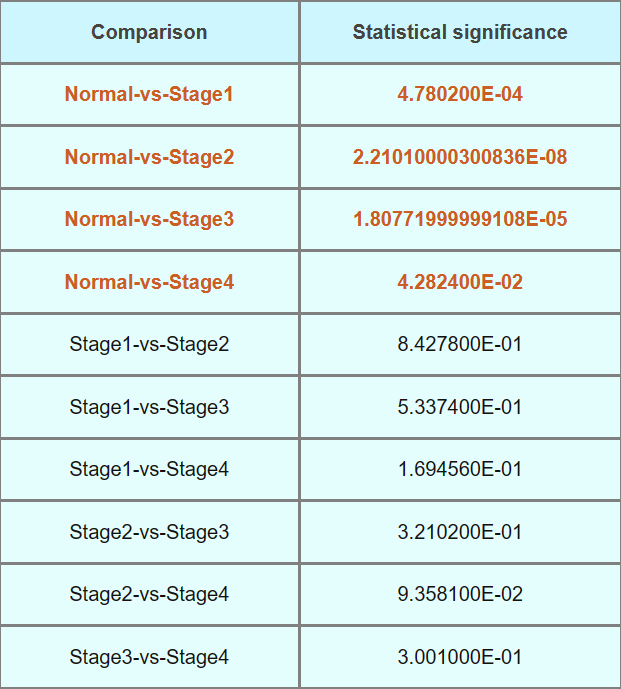


STUB1


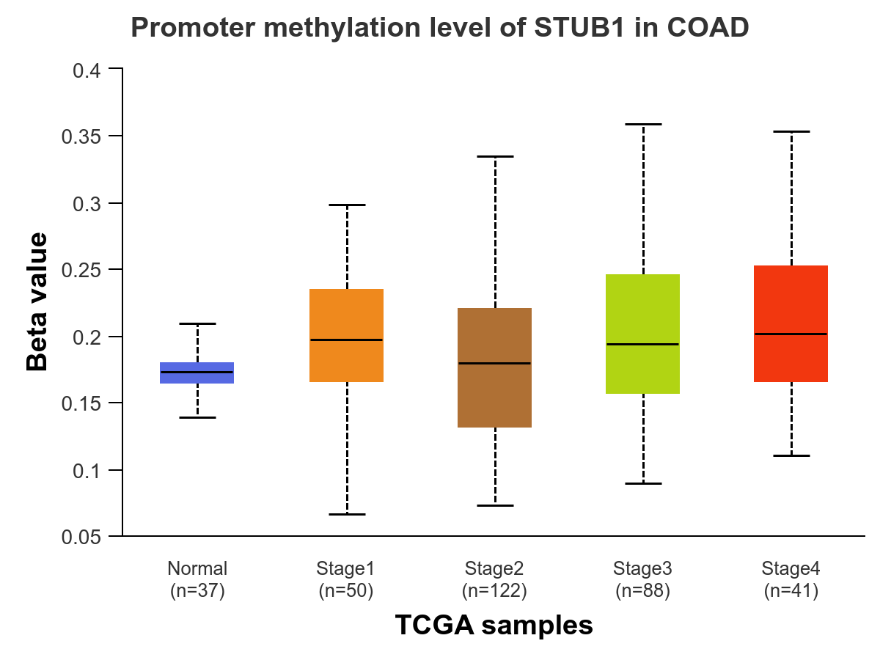


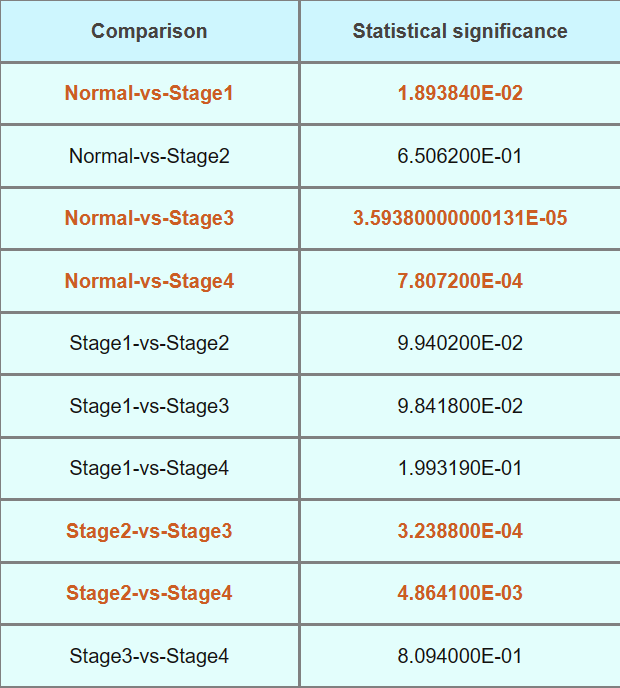


ESPL1


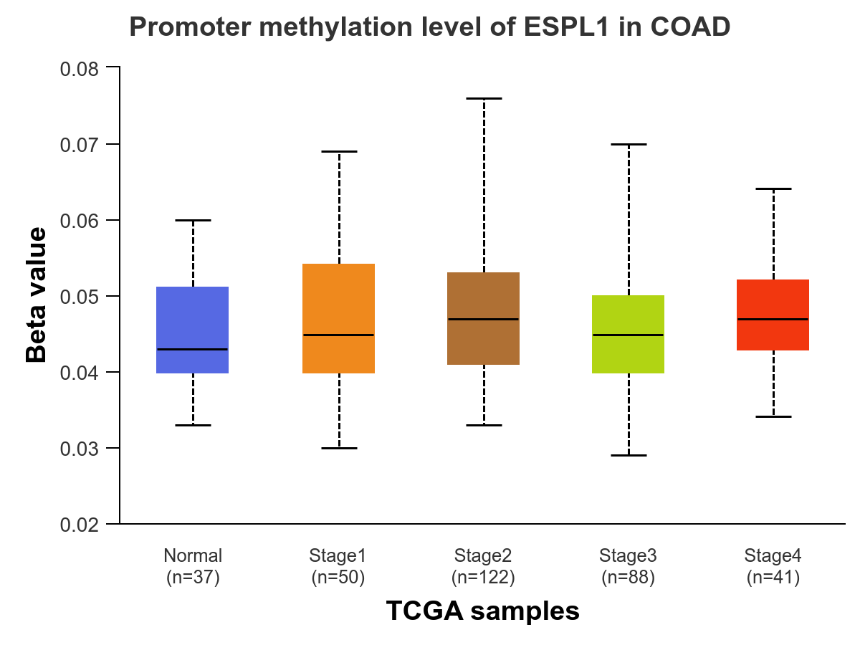


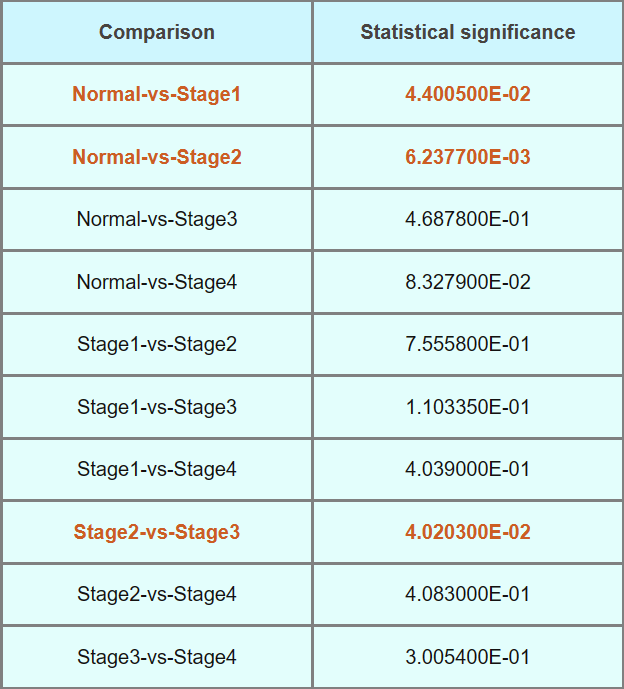


PRNP


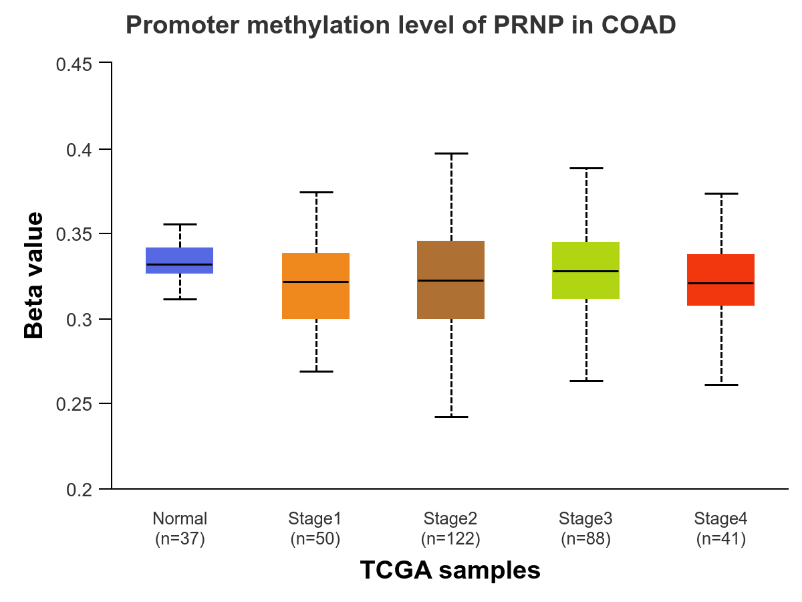


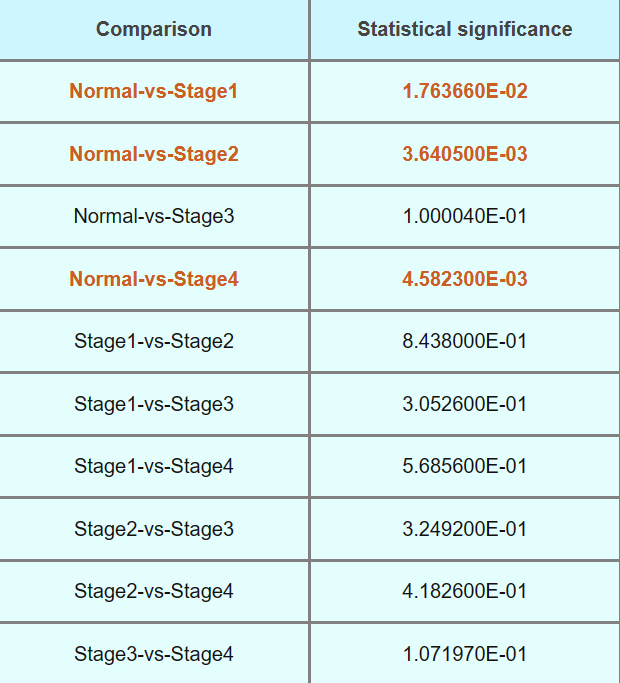


UNC45A


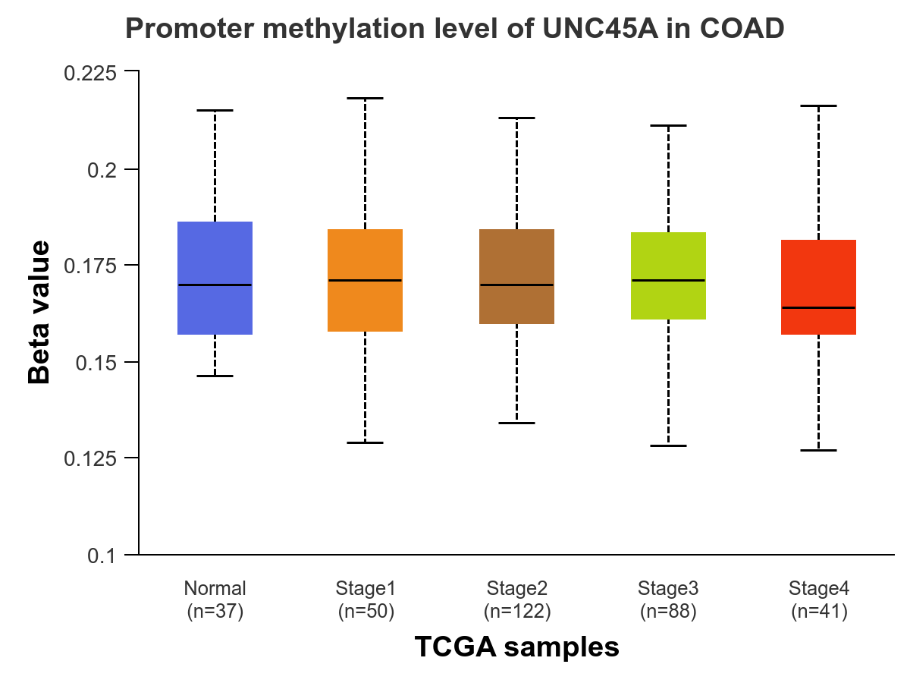


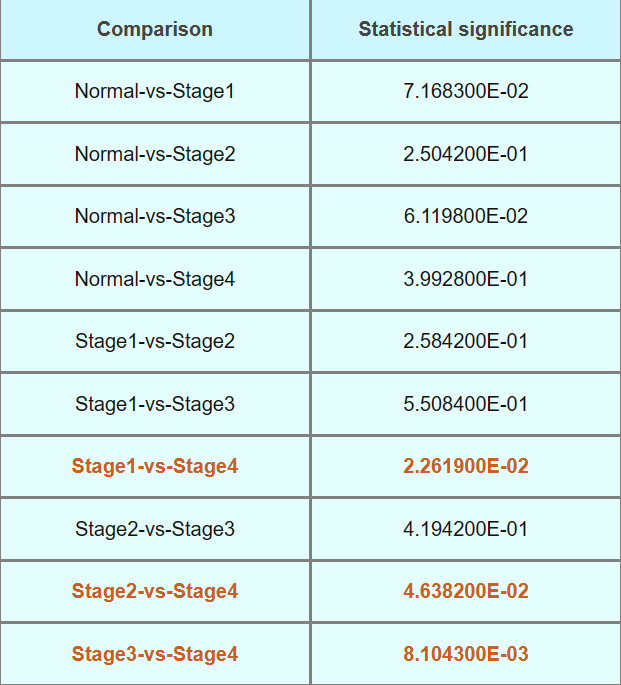


TNNI2


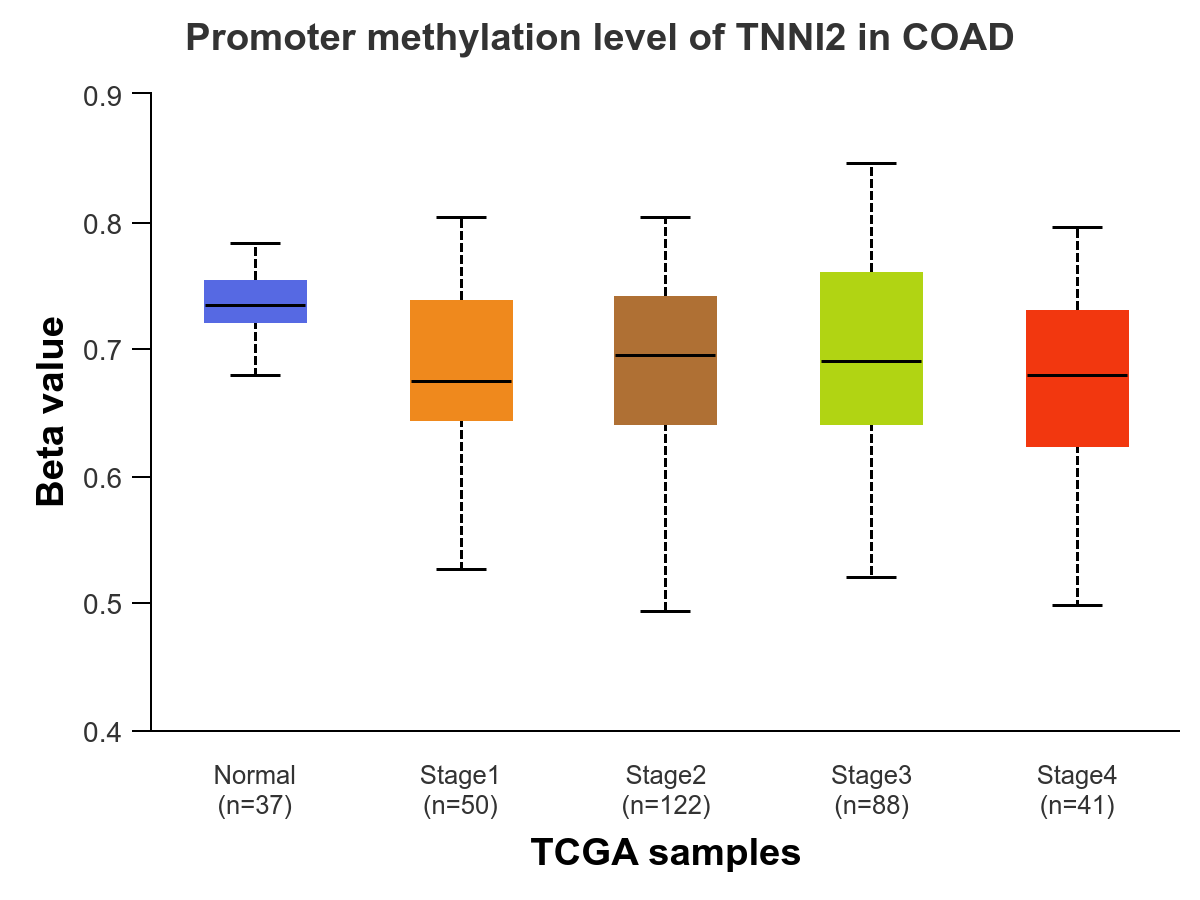


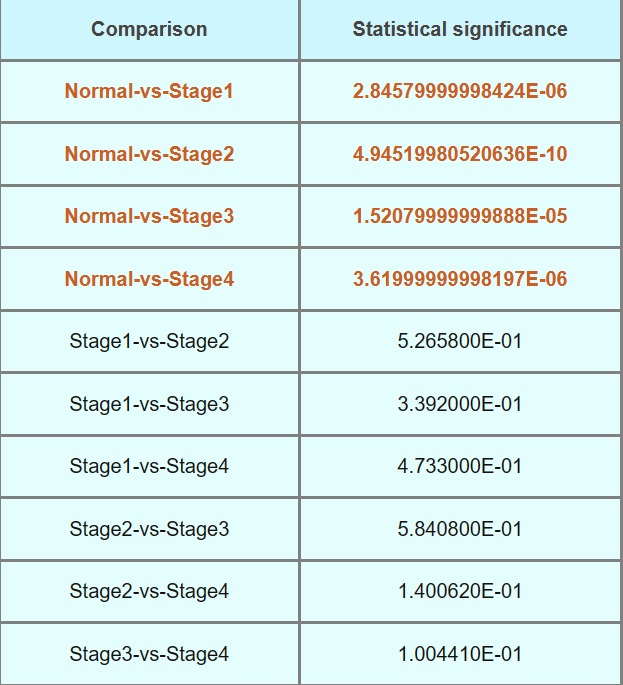


ADAMDEC1


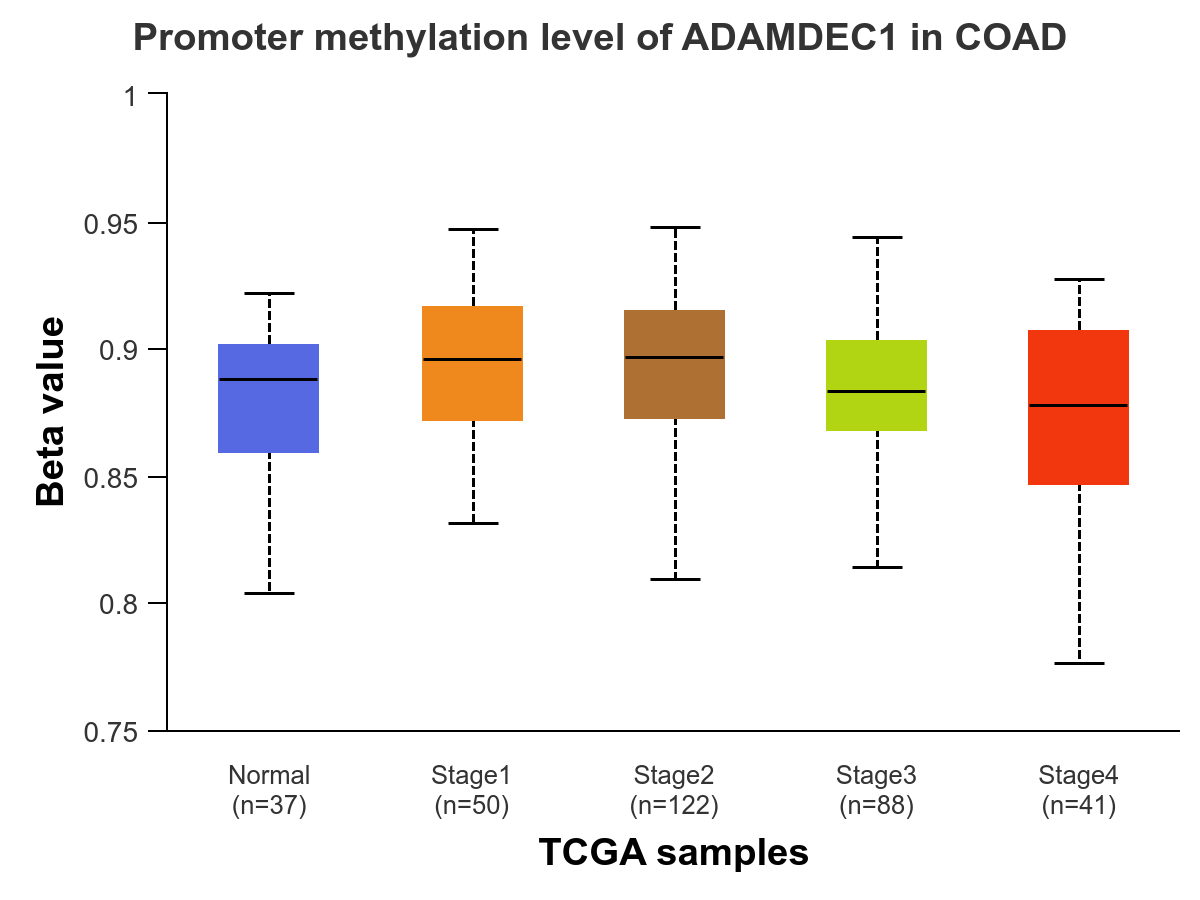


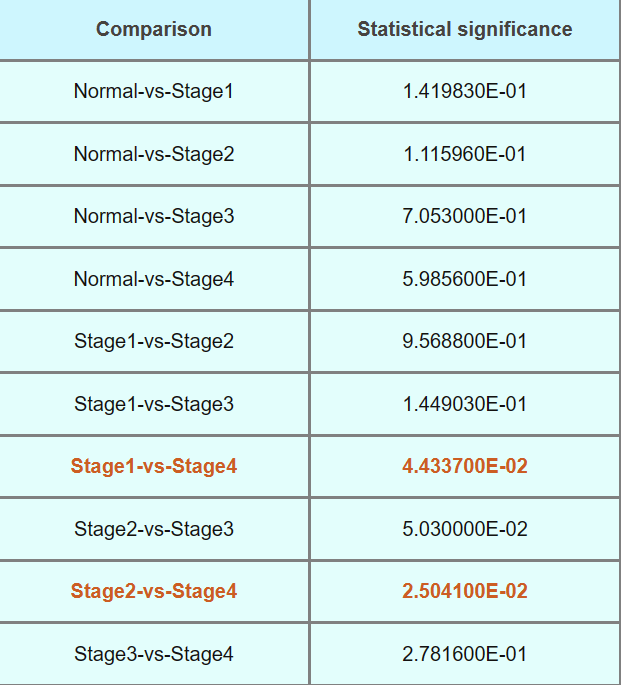

Supplement: Supplementary file 6 — Supplementary Material 6. [file 12920_2024_1898_MOESM6_ESM.docx]

cluster high low

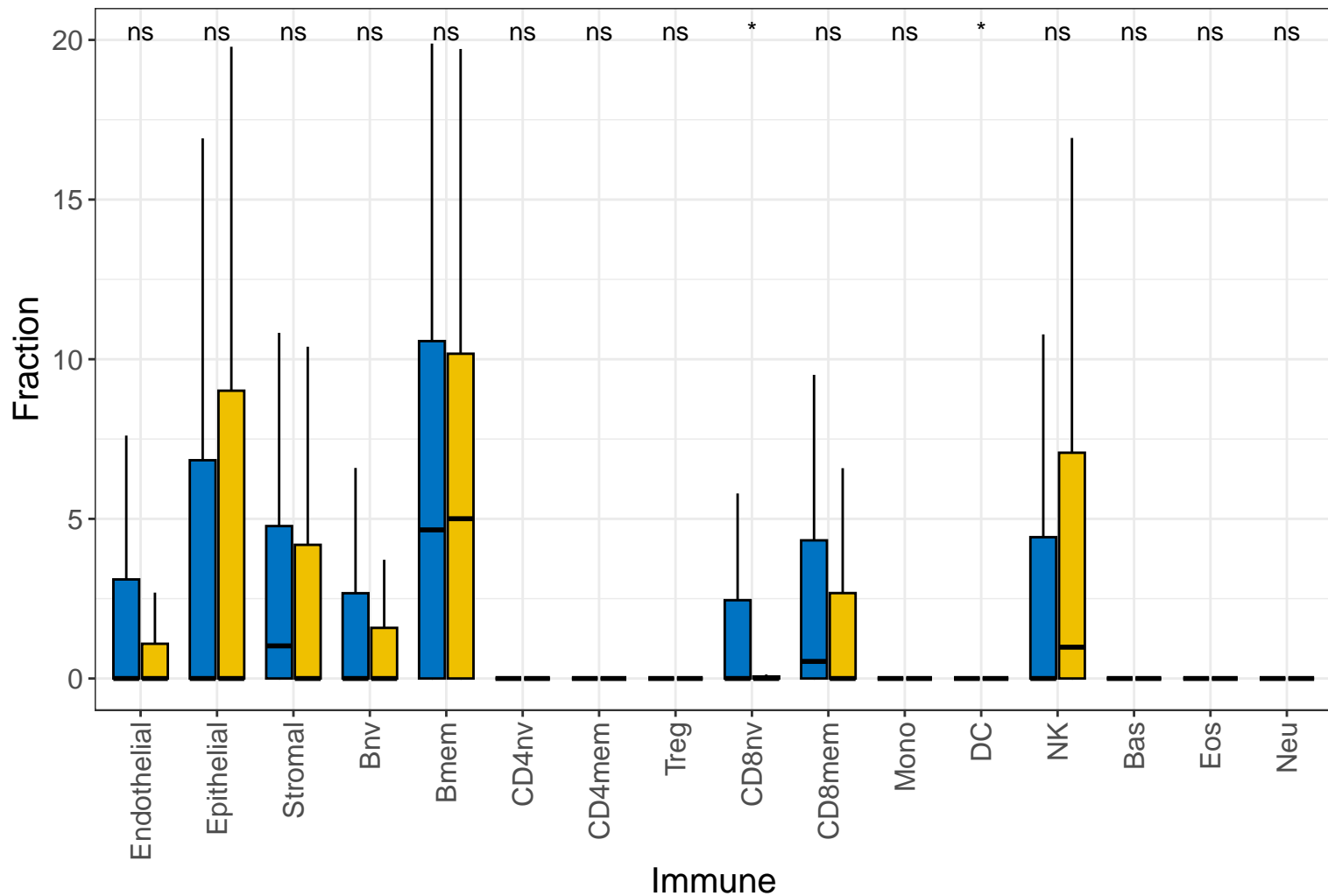

Supplement: Supplementary file 8 — Supplementary Material 8. [file 12920_2024_1898_MOESM8_ESM.pdf]
